# Integrated single-cell transcriptomics and spatial metabolomics unveil cellular differentiation and ginsenosides biosynthesis in *Panax* root tips

**DOI:** 10.1093/hr/uhaf202

**Published:** 2025-07-31

**Authors:** Lifang Yang, Zhi Yang, Mei Liu, Shuying Wang, Huanzhen Wu, Qian Yang, Luqi Huang, Ye Yang, Xiuming Cui, Yuan Liu

**Affiliations:** Faculty of Life Science and Technology, Kunming University of Science and Technology, Kunming 650000, China; Faculty of Life Science and Technology, Kunming University of Science and Technology, Kunming 650000, China; Faculty of Life Science and Technology, Kunming University of Science and Technology, Kunming 650000, China; Faculty of Life Science and Technology, Kunming University of Science and Technology, Kunming 650000, China; Faculty of Life Science and Technology, Kunming University of Science and Technology, Kunming 650000, China; Faculty of Life Science and Technology, Kunming University of Science and Technology, Kunming 650000, China; Key Laboratory of Panax notoginseng Resources Sustainable Development and Utilization of State Administration of Traditional Chinese Medicine, Kunming 650000, China; Yunnan Provincial Key Laboratory of Panax notoginseng, Kunming 650000, China; Kunming Key Laboratory of Sustainable Development and Utilization of Famous-Region Drug, Kunming 650000, China; Sanqi Research Institute of Yunnan Province, Kunming 650000, China; National Resource Center for Chinese Materia Medica, China Academy of Chinese Medical Sciences, Beijing 100700, China; Faculty of Life Science and Technology, Kunming University of Science and Technology, Kunming 650000, China; Key Laboratory of Panax notoginseng Resources Sustainable Development and Utilization of State Administration of Traditional Chinese Medicine, Kunming 650000, China; Yunnan Provincial Key Laboratory of Panax notoginseng, Kunming 650000, China; Kunming Key Laboratory of Sustainable Development and Utilization of Famous-Region Drug, Kunming 650000, China; Sanqi Research Institute of Yunnan Province, Kunming 650000, China; Faculty of Life Science and Technology, Kunming University of Science and Technology, Kunming 650000, China; Key Laboratory of Panax notoginseng Resources Sustainable Development and Utilization of State Administration of Traditional Chinese Medicine, Kunming 650000, China; Yunnan Provincial Key Laboratory of Panax notoginseng, Kunming 650000, China; Kunming Key Laboratory of Sustainable Development and Utilization of Famous-Region Drug, Kunming 650000, China; Sanqi Research Institute of Yunnan Province, Kunming 650000, China; Faculty of Life Science and Technology, Kunming University of Science and Technology, Kunming 650000, China; Key Laboratory of Panax notoginseng Resources Sustainable Development and Utilization of State Administration of Traditional Chinese Medicine, Kunming 650000, China; Yunnan Provincial Key Laboratory of Panax notoginseng, Kunming 650000, China; Kunming Key Laboratory of Sustainable Development and Utilization of Famous-Region Drug, Kunming 650000, China; Sanqi Research Institute of Yunnan Province, Kunming 650000, China

## Abstract

Root tips, which represent the initial stage of taproot development, serve as an ideal model for investigating plant growth and secondary metabolism. However, studies of root tips in *Panax* species have been limited, restricting our understanding of cell fate transitions during early root development and the cellular heterogeneity associated with ginsenosides biosynthesis. To address this gap, we conducted single-cell RNA sequencing (scRNA-seq) and spatial metabolomics analyses on the root tips of three *Panax* species: *Panax notoginseng*, *Panax ginseng*, and *Panax quinquefolium*. Our research reconstructed the developmental trajectory of the early endodermis and revealed epidermis-specific expression patterns of key enzyme genes involved in ginsenosides biosynthesis. We identified several novel transcription factors (TFs): *IAA29* (which positively regulates endodermis suberization) and *MYB2*/*MYB78* (positive regulators of ginsenosides biosynthesis), validated by dual-LUC reporter and electrophoretic mobility shift assay (EMSA). Conserved and divergent ligand-receptor interaction patterns across the three *Panax* species were discovered, with the *FAD* gene family exhibiting tissue- and species-specific expression. Cell-specific genes expression was confirmed by RNA *in situ* hybridization. Mass spectrometry imaging (MSI) mapped ginsenosides spatial distribution, while LC–MS/MS verified species-specific biosynthesis. This study presents a single-cell transcriptional landscape of early differentiation and cell type-specific ginsenosides accumulation in the *Panax* genus.

## Introduction

The *Panax* genus comprises approximately 20 species or variants, with *P. notoginseng* (PN), *P. ginseng* (PG), and *P. quinquefolium* (PQ) representing the most economically significant taxa due to their medicinal value [1, 2]. PG and PQ form a sister clade that diverged from the PN lineage approximately 5 million years ago, offering distinct evolutionary models [3, 4]. These species exhibit characteristic metabolic profiles: PN accumulates high levels notoginsenoside R_1_ and dammarane-type saponins (e.g., Rg_1_ and Rb_1_); PG is rich in dammarane-types (e.g., Rg_1_, Re, and Rb_1_) with trace amounts of oleanane-types (e.g., Ro); whereas PQ produces unique pseudoginsenoside F11 and displays elevated Rb_1_/Rg_1_ ratios [2]. Together, these three species accounting for more than 90% of global ginseng production, enabling comparative analyses of conserved and species-specific mechanisms underlying root development and specialized metabolism.

The taproot serves as the primary medicinal organ in *Panax* species, while the root tip orchestrates taproot development through spatially organized differentiation. Within this critical zone, cell subtypes along the root apex-to-base axis exhibit a longitudinal differentiation trajectory [5]. Particularly, the endodermis—surrounding the vasculature—functions as a selective barrier regulating nutrient transport in higher plants [6]. Critically, establishing such functional barriers partially relies on cell–cell communication (CCC) networks coordinating cell fate decisions through ligand-receptor signaling [7]. Therefore, elucidating CCC networks, endodermal differentiation trajectory, and associated transcriptional regulatory mechanisms is essential for understanding development plasticity in *Panax* root tips.

Ginsenosides, pharmacologically active triterpenoid saponins unique to *Panax*, accumulate predominantly in roots [8]. While bulk RNA-seq and metabolomics have examined their biosynthesis in individual species [9–14], cellular-resolution gene-metabolite coordination remains poorly characterized [14, 15]. Notably, spatial heterogeneity in ginsenoside distribution and its relationship with cell type-specific expression of biosynthetic genes have been unresolved.

ScRNA-seq has revolutionizes transcriptomic studies by resolving genes expression at cellular resolution. This technology has elucidated diverse biology processes, including cell fate determination [16], stomata development [17], root development and metabolism [18, 19], inflorescence development and fruit senescence [20, 21], fiber initiation [22, 23], xylem formation [24, 25], root nodulation [26], symbiotic interactions [27], and stress responses [28, 29]. Crucially, scRNA-seq enable spatial mapping of biosynthetic genes, revealing cell type-specific metabolite production [30–32]. The application of scRNA-seq in non-model plants remains limited due to the lack of reference genomes and technical challenges associated with protoplasting. To date, scRNA-seq has been applied to model plants, such as *Arabidopsis thaliana*, and major crops, including *Oryza sativa*, *Zea mays*, and *Gossypium* spp*.* [33–39], and only a few medicinal plants, such as *Catharanthus roseus*, *Taxus mairei*, *Nepeta tenuifolia*, and *Limonium bicolor*, have been investigated [30, 31, 40, 41]. Complementarily, spatial metabolomics emerges as a powerful tool for mapping spatial distributions of metabolites. By precisely visualizing compounds localization, MSI provides mechanistic insights into biosynthetic pathways [42]. This approach has successfully characterized bioactive compounds in medicinal plants, such as *Taxus mairei*, *Dendrobium nobile*, *Salvia miltiorrhiza*, and *Catharanthus roseus*, but has not been integrated with scRNA-seq in *Panax* [42].

Herein, we integrate scRNA-seq and spatial metabolomics to investigate cell differentiation and ginsenosides biosynthesis in the root tips of PN, PG, and PQ. Our study: (i) reconstructs endodermis differentiation trajectories, (ii) maps ginsenosides heterogeneity using MALDI2-MSI, (iii) quantifies species-specific ginsenosides using LC–MS/MS, and (iv) identifies transcriptional regulators involved in cellular differentiation and ginsenosides biosynthesis. Furthermore, we characterize conserved and divergent ligand-receptor (L–R) interactions as well as tissue- and species-specific expression patterns of the *FAD* gene family. This multi-omics approach provides insights into *Panax* root development and specialized metabolism, offering a foundation for precision breeding.

## Results

### Construction of a single-cell atlas for three *Panax* root tips

**Figure 1:**
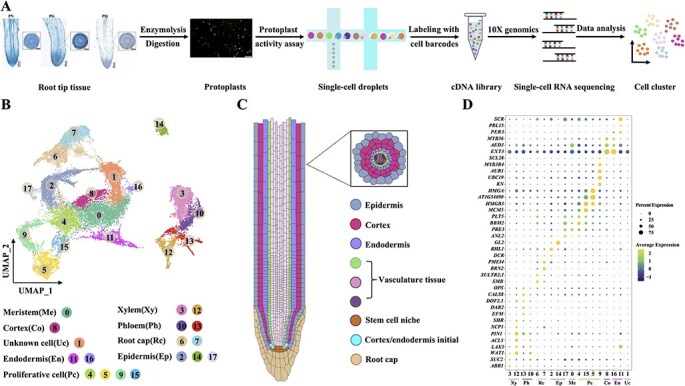
Generation of a single-cell RNA sequencing (scRNA-seq) atlas for the root tips of three *Panax* species. (A) The workflow for scRNA-seq of the root tips from three *Panax* species is illustrated. Root tips were stained with safranin O-fast green. Vertical scale bars represent 500 μm, while horizontal scale bars denote 100 μm. **(**B) UMAP visualization depicting 18 distinct clusters derived from a total of 24 425 cells. Each dot represents an individual cell with colors indicating their respective cell clusters. (C) Anatomy schematic showing both longitudinal section (left) and cross-sectional (right) views of the root tip anatomy. (D) DotPlot illustrating 39 cell type-specific marker genes; the diameter of each dot reflects the percentage of cells expressing a given marker gene within its corresponding cluster, while dot color indicates the average scaled expression level of that marker gene in the cluster (with yellower hues representing higher expression levels and bluer hues indicating lower expression levels). The full names of all marker genes were provided in Table S5. Xy, xylem; Ph, phloem; Rc, root cap; Me, meristem; Pc, proliferative cell; Co, cortex; En, endodermis; Uc, unknown cell.

Following seed germination, root tip samples were enzymatically digested to isolate protoplasts. Single-cell libraries were constructed using the 10× Chromium platform (10× Genomics, Pleasanton, CA, USA) (Fig. 1A, Fig. S1). After filtering empty droplets, we obtained 6036, 7424, and 12 837 single cells for PN, PG, and PQ, respectively (Table S1). To evaluate the robustness of the scRNA-seq results, each dataset was randomly split into two subsamples, which exhibited high consistency between the two subsamples for PN, PG, and PQ (Fig. S2A). Pearson correlation coefficients between bulk RNA-seq (Table S2) and scRNA-seq data were 0.86 (PN), 0.88 (PG), and 0.89 (PQ) (Fig. S2B). Post quality control (Fig. S3)—excluding low-expression genes, low-quality cells, and doublets—5761 (PN), 7026 (PG), and 11 638 (PQ) high-confidence single cells were retained. Expression levels of protoplasting-related gene per cell cluster were quantified and visualized (Fig. S4A). Cell clustering patterns remained consistent before and after removing these genes (Fig. S4B), confirming their negligible impact on clustering. Batch effects among PN, PG, and PQ were corrected using Harmony v0.1.1 package (Fig. S5A) [43]. Integration of the three datasets yielded a 24 425 cells × 32 254 gene expression matrix for downstream analysis.

**Figure 2:**
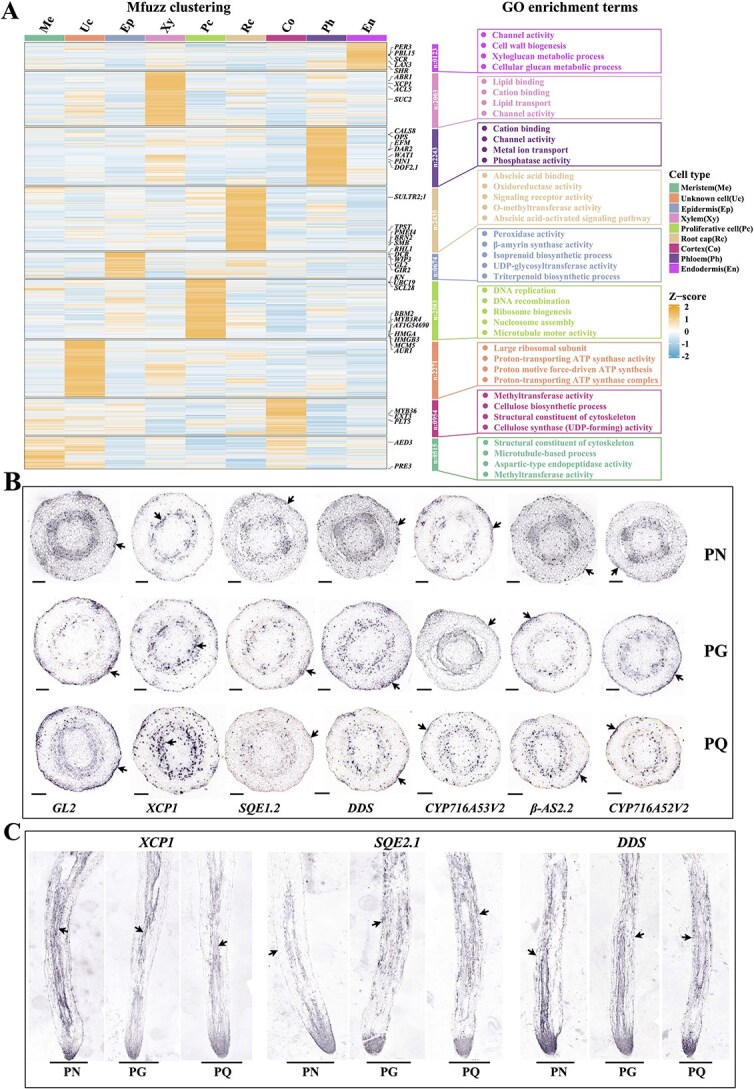
The clustering patterns of all genes and the experimental results of RNA *in situ* hybridization. (A) Dynamic expression patterns for all detected genes based on Mfuzz algorithm and GO function enrichment analyses for differentially expressed genes across various cell type. Genes exhibiting similar expression dynamics have been clustered into specific groups, with known cell type marker genes highlighted on the heatmap. GO function enrichment results pertaining to differentially expressed genes within each cell type are displayed to the right side of the heatmap. (B) Cross sectional view showing RNA *in situ* hybridization*.* (C) Longitudinal section displaying findings from RNA *in situ* hybridization.

To visualize cell clustering, we selected 2000 highly variable genes and applied both uniform manifold approximation and projection (UMAP) algorithms and *t*-distributed stochastic neighborhood embedding (*t*-SNE) for dimensional reduction (Fig. 1B, Fig. S5B). Graph-based clustering analysis (resolution = 0.6) identified 18 transcriptionally distinct cell populations among 24 425 single cells, as determined using the FindClusters function in Seurat (Fig. 1B, Fig. S5C). Analysis of cell cycle genes revealed the similar proportions of cell phase (G1 > S > G2M) across all clusters except for cluster 5, 9, 15 (Fig. S5D). Cluster-specific marker genes (Table S3) and cluster-enriched genes (Table S4) were identified by analyzing differentially expressed genes (DEGs) among the 18 clusters. For accurate annotation, known marker genes with their verified expression patterns and biological functions from plant scRNA-seq datasets were used to identify cell types (Table S5). The 18 clusters were categorized into nine different cell types: epidermis (Ep), root cap (Rc), endodermis (En), cortex (Co), xylem (Xy), phloem (Ph), meristem (Me), proliferative cell (Pc), and unknown cell (Uc) (Fig. 1B and C, Table S6). Clustering analysis using the Mfuzz algorithm indicated that all genes were grouped into nine clusters, with genes within the same cluster exhibiting similar expression dynamics, was consistent with the cell type identification based on known markers (Fig. 2A).

Overall, the root tips of the three *Panax* species exhibited highly heterogeneous cellular compositions. Thirteen known cell type marker genes showed exclusive expression in vascular populations (Fig. 1D). For instance, *ABA REPRESSOR1* (*ABR1*), a marker for xylem parenchyma cells, was highly expressed in cluster 3 [29, 44]. *ACL5* (*ACAULIS 5*), predominantly localized to xylem, displayed specific enrichment in cluster 12 [32, 36]. *WAT1* (*WALLS ARE THIN 1*), associated with xylem fiber development, was preferentially expressed in clusters 10 and 12 [45]. *CALS8* (*CALLOSE SYNTHASE 8*), a marker for phloem pole pericycle, along with *EFM* (*EARLY FLOWERING MYB PROTEIN*) and *DAR2* (*DA1-RELATED PROTEIN 2*), markers for phloem companion cells, and *OPS* (*OCTOPUS*), a marker for protophloem formation, were co-enriched in cluster 13 [29, 44, 46, 47]. *XCP1*(*XYLEM CYSTEINE PEPTIDASE 1*), marking sieve and tracheary elements, peaked in cluster 12 [32]. *GL2* (*GLABRA 2*), an epidermal differentiation marker, was predominant in cluster 14 [5, 48, 49]. Epidermal markers *RHL1* (*ROOT HAIRLESS 1*), *DCR* (*DEFECTIVE IN CUTICULAR RIDGES*), and *PMEI4* (*PECTINMETHYLESTERASE INHIBITOR 4*), localized to clusters 2 and 17 [33, 34]. *SMB* (*SOMBRERO*), a root cap-specific marker, was enriched in clusters 6 and 7 [50]. Cluster 0 exhibited dominant expression of *PRE3* (*PACLOBUTRAZOL RESISTANCE 3*, *TMO7*) and *PLT5* (*PLETHORA 5*), crucial for meristem maintenance [51–53]. Clusters 4, 5, and 15 showed enrichments of *MCM* (*MINICHROMOSOME MAINTENANCE*), *HMGB* (*HIGH MOBILITY GROUP B*), and *HIS2A* (*Histone H2A*), implicating active cell division and proliferation [28, 32, 46]. Cluster 9 contained cell-cycle genes, including *CDKB2;2* (*CYCLIN-DEPENDENT KINASE B2;2*), *CYCB2;3* (*CYCLIN B2;3*), *AT5G25380* (*CYCA2;1*), *CYCA1;1* (*CYCLIN A1;1*), and *SCL28* (*SCARECROW-LIKE 28*), as well as cycle-related genes such as *KN* (*KNOLLE*), *AUR1* (*ATAUR 1*), *UBC19* (*UBIQUITIN-CONJUGATING ENZYME19*), and *AT5G11510* (*MYB3R4*) [28, 32, 53]. Based on phase-specific markers, clusters 4, 15, 5, and 9 were annotated as G0/G1, S, G2, and M phases proliferative cell, respectively (Fig. S6A and B, Table S7). Endodermal markers *PER3* (*PEROXIDASE 3*) and *PBL15* (*PBS1-LIKE 15*) were restricted to cluster 11 [5, 48], while *MYB36* (*MYB DOMAIN PROTEIN 36*, casparian strip regulator) localized to clusters 8 and 16 [54, 55]. Cortical markers *EXT3* (*EXTENSIN 3*) and *AED3* (*APOPLASTIC EDS1-DEPENDENT 3*) were unique to cluster 8 [50]. Cluster 16 enriched *SHR* (*SHORT ROOT*) and *SCR* (*SCARECROW*), marking cortex/endodermis asymmetric division initial [35]. Cluster 1 lacked known marker and was designated unknown cells (Fig. 1D).

Cell- type annotations were validated through Mfuzz clustering, GO enrichment, and KEGG analyses (Fig. 2A, Figs S6C and S7, Table S7). Vascular GO terms highlighted channel activity, ion transport, and lipid trafficking, aligning with xylem/phloem functions. Epidermal clusters were enriched in triterpenoid biosynthesis, β-amyrin synthase, and UDP-glycosyltransferase (UGT) activity, suggesting epidermis-specific ginsenosides accumulation. Root cap terms ‘abscisic acid binding’ and ‘ABA-activated signaling’ reflected its limited division capacity via cell cycle suppression. RNA *in situ* hybridization confirmed tissue-specific expression: *GL2* and key ginsenoside biosynthetic enzyme genes—including *SQE* (squalene epoxidases), *β-AS* (β-amyrin synthase), *CYP716A52V2* (β-amyrin 28-oxidas*e*), *DDS* (dammarenediol synthase), and *CYP716A53V2* (protopanaxatriol synthase)—were localized to the epidermis, while *XCP1* expression was vascular-specific (Fig. 2B and C, Fig. S8). Additionally, several cell type-specific marker genes with elevated expression in *Panax* species were identified (Fig. S9, Table S5).

### Dynamic gene expression during endodermis development

**Figure 3:**
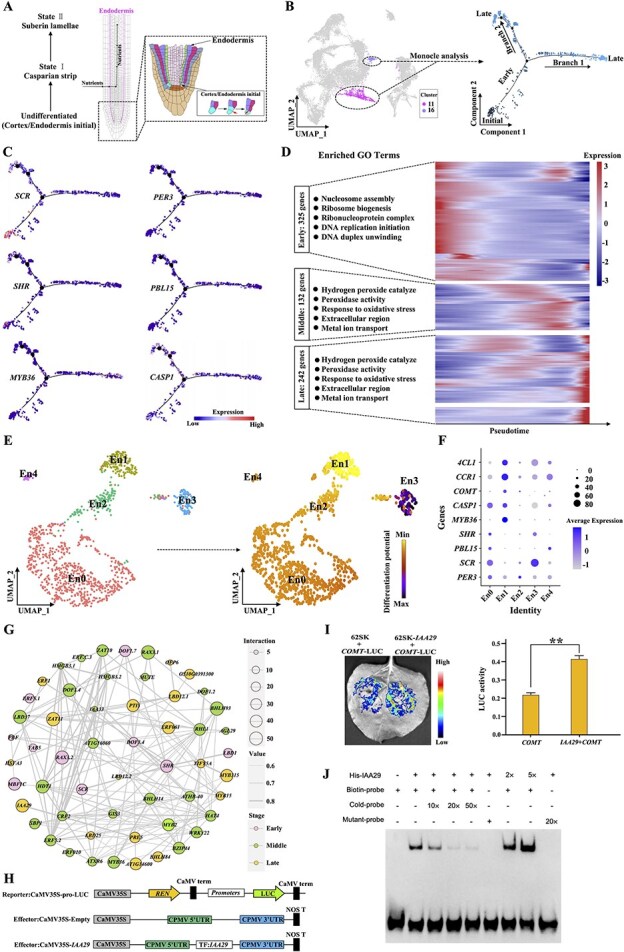
Transcriptional dynamics during endodermis development. (A) Schematic diagram depicting the developmental trajectory of the endodermis. (B) UMAP plot (consistent with Fig. 1B) highlighting only endodermal cells and their Pseudotime development trajectory constructed using Monocle2. Each point represents a unique cell, with color changes (from dark to light) indicating the trajectory of endodermis development (from early to late stages). (C) Dynamic expression profiles of key genes involved in endodermis development along the pseudotime trajectory. Dot color represents the expression level of the corresponding gene in an individual cell. (D) Pseudotime heatmap showing log-normalized expression levels of 698 genes dynamically expressed during endodermis development. Left panel: top five significantly enriched GO terms at each stage. (E) UMAP plot of sub-clusters within the endodermal cell population and estimation of endodermis differentiation trajectory using Palantir. The color bar represents differentiation potential, with each dot representing a single cell and different colors denoting different sub-clusters. (F) Expression patterns of key genes involved in endodermis differentiation. Dot diameter indicates the percentage of cells expressing a given gene within a sub-cluster, and dot color represents the average scaled expression level of the gene in that sub-cluster. Darker colors indicate higher expression level. (G) Transcriptional regulatory network constructed using 55 TFs dynamically expressed across the endodermis differentiation trajectory. Node size reflects the number of interactions, line width corresponds to correlation values, and three colors represent three developmental stages. (H) Promoter fragment was cloned into pGreenII 0800-LUC to generate reporter construct, and effector construct was produced by inserting *IAA29* into pGreenII-62-SK. (I) Overview and results of dual-luciferase reporter gene assays. Relative LUC activity represents the activity ratio of firefly luciferase to Renilla luciferase. Values are means ± standard deviation from three biological replicates, ^**^p < 0.01. (J) The his-*IAA29* fusion protein was incubated with probe containing the binding element derived from the promoter of *COMT* gene. − and + represent absence and presence, respectively. 2× or 5× show increasing amounts of fusion proteins, and 10×, 20×, or 50× show increasing amounts of probes for competition.

Theoretically, distinct subpopulations within a specific cell type can reflect the trajectory of cell differentiation. The endodermis undergoes a differentiation process from an undifferentiated state (cortex/endodermis initials) to State I (casparian strip formation), and then to State II (suberin formation) (Fig. 3A) [5]. Therefore, all 1117 cells identified as endodermis were utilized for the pseudo-time analysis (Fig. 3B). A total of 698 genes were found to be dynamically expressed along the endodermal differentiation trajectory and were categorized into three expression stages: early, middle, and late stages, based on the pseudo-time trajectory (Table S8). To accurately depict the pseudo-time axis corresponding to the endodermal differentiation process in the *Panax* genus, six typical endodermal marker genes were mapped onto the pseudo-time trajectory (Fig. 3C). The early stage included *SHR* and *SCR*, known TFs that regulate asymmetric division of the cortex/endodermis initials [35, 56, 57]. Genes highly expressed in this stage were enriched in DNA replication and nucleosome assembly functions. Genes highly expressed in the middle stage included *PER3* and *PBL15*, which are known to regulate endodermis differentiation [18, 56, 58]. Enriched GO functions in this stage were related to hydrogen peroxide catabolic process and peroxidase activity. Genes with late expression peaks included *CASP1* and *MYB36*, typical markers associated with casparian strip formation. Enriched GO functions in this stage were concentrated on xyloglucan metabolic processes and xyloglucosyl transferase activity (Fig. 3D). Subpopulation analysis revealed that endodermal cells could be further divided into five subpopulations. Analysis of the differentiation potential indicated that subpopulation En3 exhibited the greatest differentiation potential while En1 had the least. Subpopulation En0, En2, and En4 were in transitional states of differentiation (Fig. 3E). *SCR* and *SHR*, which regulate asymmetric division of cortex/endodermis initials, were highly expressed in subpopulation En3, corresponding to the early stage of pseudo-time development; *MYB36* and *CASP1*, markers related to casparian strip formation, along with *4CL1* (*4-COUMARATE: COA LIGASE 1*), *CCR1* (*CINNAMOYL COA REDUCTASE 1*), and *COMT* (*CAFFEATE O-METHYLTRANSFERASE*), which are involved in suberin biosynthesis, were enriched in En1, corresponding to the late stage of pseudo-time development; *PER3* and *PBL15*, which regulate endodermal differentiation, were specifically expressed in En0, En2, and En4, corresponding to the middle stage of the pseudo-time trajectory (Fig. 3F, Fig. S10A). RNA velocity analysis confirmed that subpopulation En3 is the origin of endodermal differentiation, exhibiting maximum differentiation potential, while En1 is in the late stage of differentiation, showing minimum differentiation potential. The remaining subpopulations (En0, En2, and En4) were in transitional states (Fig. S10B). Additionally, staining observations confirmed the presence of slight casparian strips and suberin in PN, PG, and PQ root tips, validating the differentiation process of the endodermis, followed by suberin observation in the epidermis (Fig. S11).

The specific TRNs that directly regulate suberin biosynthesis have not been well identified [5]. To further elucidate the transcriptional regulation responsible for suberization in *Panax* endodermis, we constructed a TRN comprising 55 TFs that are dynamically expressed along the endodermis development trajectory. The result indicated that 17 TFs participated in regulating late endodermis differentiation (Table S9). Among these, TFs *IAA29* (*INDOLE-3-ACETIC ACID INDUCIBLE 29*), *MYB315* (*MYB DOMAIN PROTEIN 315*), and *ZAT11* (*ZINC FINGER OF ARABIDOPSIS THALIANA 11*), which belong to the late-expression category, stand out as positive regulators of suberin biosynthesis. This suggested their potentially strong involvement in regulating root tip endodermis suberization in the *Panax* species (Fig. 3G). Transient dual-luciferase reporter gene assay and electrophoretic mobility shift assays confirmed that *IAA29* directly regulates the expression of *COMT* genes related to suberin and lignin biosynthesis (Fig. 3H–J). Overall, these findings highlighted the potential of scRNA-seq in generating hypotheses that may bring novel insights into plant cellular differentiation.

### Both conserved and divergent L–R interactions among the three *Panax* species

L–R pairs were predicted through homologous alignment using PlantPhoneDB [5]. We identified 444 (57 autocrine and 387 paracrine), 401 (48 autocrine and 353 paracrine), and 477 (59 autocrine and 418 paracrine) significant L–R pairs between pairwise cell types in PN, PG, and PQ, respectively (Table S10). CCC strength was quantified by the number of interacting L–R pairs [5]. Autocrine signaling was the strongest in cortex-cortex interactions, while the most robust paracrine signaling occurred between endodermis and cortex (Fig. S12A and B). Key L–R pairs—*THE1* (*THESEUS1*), *FER* (*FERONIA*), *MPK3* (*MITOGEN-ACTIVATED PROTEIN KINASE 3*), *AT5G24010*, *HERK1* (*HERCULES RECEPTOR KINASE 1*), and *SKU5—*emerged as central regulators of CCC in *Panax* species (Fig. S12C). These predominantly kinase-associated proteins function in plant growth pathways: *THE1* and *HERK1* are brassinosteroid-regulated, while *MPK3* mediates stress responses. Expression distribution analysis of the top 10 L-R pairs (ranked by average score) revealed that all pairs were detected across PN cell–cell interactions. Among these, *UBQ3*-*FER* and *UBQ3*-*THE1* showed high expression in all PN cell pairs. PG and PQ exhibited highly similar L–R expression patterns, except for specific localization of *LRX3*-*FLA2* (Fig. S13A). Proliferative cells dominated all three species' cell populations, Ro/e (ratio of observed to expected) analysis indicated strong cell type preference in PQ endodermis/cortex and PN root cap (Fig. S13B and C).

### Cell heterogeneity and species specificity in ginsenoside biosynthesis

**Figure 4:**
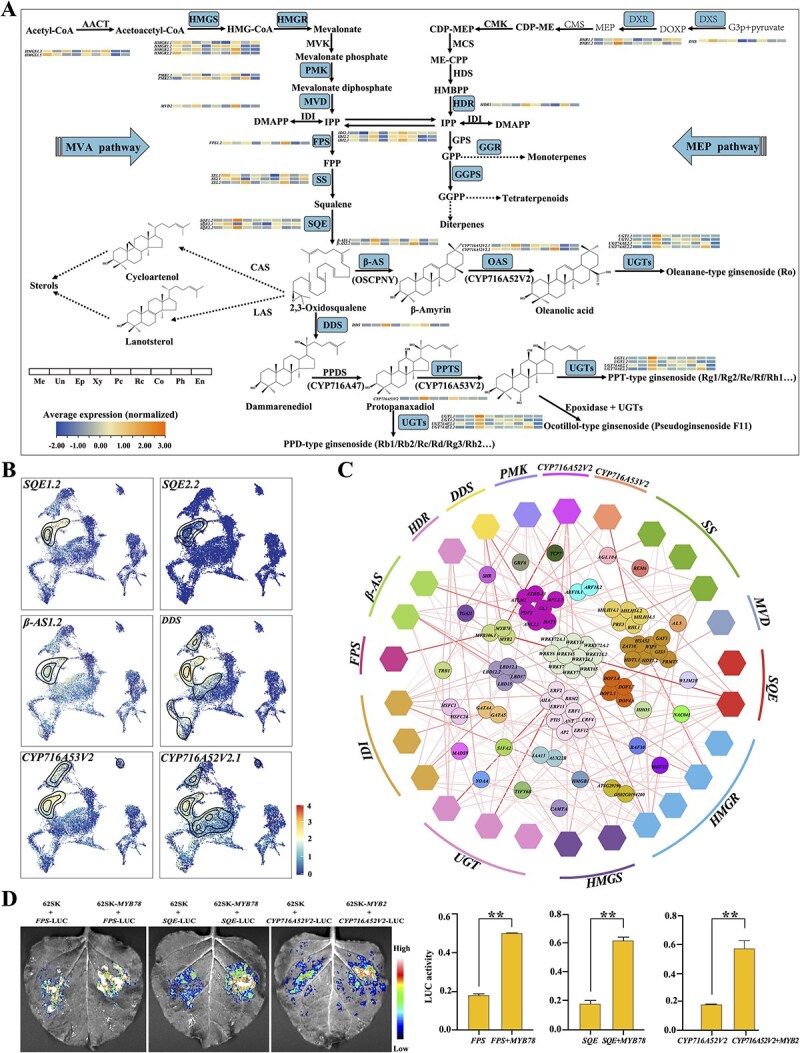
Biosynthesis pathways of ginsenosides in the *Panax* genus, as well as expression profiles and transcriptional regulation of key enzyme genes. (A) Schematic diagram of ginsenosides biosynthesis pathways in the *Panax* genus. (B) Cell type heterogeneity in the expression of key enzyme genes involved in downstream ginsenosides biosynthesis pathways. (C) Correlation analysis between key enzyme genes responsible for ginsenosides biosynthesis pathways and transcription factors. Transcription factors were represented by circles, and enzyme genes by hexagons. Lines indicate the nature of correlation between transcription factors and enzyme genes, with darker colors signifying stronger correlations (correlation cutoff ≥0.7). (D) Overview and results of dual-luciferase reporter gene assays. Relative LUC activity represents the activity ratio of firefly luciferase to Renilla luciferase. Values are means ± standard deviations from three biological replicates, ^**^p < 0.01.

Key enzymes involved in ginsenoside biosynthesis have been extensively characterized and reported in *Panax* species [1]. In our scRNA-seq datasets, we identified numerous genes encoding enzymes involved in both up- and downstream pathways of ginsenoside biosynthesis. Specifically, upstream pathway genes, including five *HMGS* (hydroxymethylglutaryl-CoA synthase), eight *HMGR* (hydroxymethylglutaryl-CoA reductase), six *PMK* (phosphomevalonate kinase), one *MVD* (mevalonate diphosphate decarboxylase), two *FPS* (farnesyl diphosphate synthase), five *SS* (squalene synthase), three *IDI* (isopentenyl diphosphate isomerase), one *DXS* (deoxy-d-xylulose phosphate synthase), two *DXR* (deoxy-d-xylulose phosphate reductoisomerase), and one *HDR* (hydroxymethyl butenyl pyrophosphate reductase) gene, were present in multiple copies. Downstream pathway genes included five *SQE* (squalene epoxidases), six *β-AS* (β-amyrin synthase), two *CYP716A52V2* (β-amyrin 28-oxidase), one *DDS* (dammarenediol synthase), one *CYP716A53V2* (protopanaxatriol synthase), and six *UGT* genes (Fig. 4A). Among these, *HMGS1.3*, *HMGS1.5*, *HMGR1.1*, *HMGR2.1*, *PMK1.1*, *MVD2*, *FPS1.2*, *SS1.1*, *SS2.2*, *SQE1.2*, *SQE2.2*, *DDS*, *IDI2.2*, *IDI2.3*, *CYP716A53V2*, *CYP716A52V2.2*, *β-AS2.2*, *UGT1.1*, *UGT1.2*, *UGT74AE2.1*, and *UGT74AE2.2* exhibited the highest expression levels across the largest proportion of cells (Fig. S14). Further analysis revealed that most ginsenoside biosynthesis-related genes, particularly those involved in downstream pathways, were specifically overexpressed in epidermal cells (clusters 2, 14, and 17) (Fig. 4B, Fig. S15A). In the downstream pathways, both *DDS* and *CYP716A53V2*, which are responsible for the dammarane-type ginsenosides biosynthesis, showed higher expression levels in PN than in PG and PQ. Conversely, *β-AS* and *CYP716A52V2*, which are responsible for oleanane-type ginsenosides biosynthesis, exhibited higher expression levels in PG and PQ than in PN (Fig. S15B). This expression pattern was consistent with findings from bulk RNA-seq datasets (Fig. S16).

We integrated the three *Panax* species and further dissected the TRNs involved in ginsenoside biosynthesis. A total of 2675 TFs from 88 different families were identified in our datasets (Table S11), with 92 TFs specifically and highly expressed in epidermal cells (Table S12). We mapped the TRNs between these 92 differentially expressed TFs and enzyme genes involved in ginsenoside biosynthesis pathways using correlation analysis (Fig. 4C). The results indicated that the *WRKY*, *ERF*, and *C2H2* families are extensively involved in ginsenoside biosynthesis. Among the 92 TFs, *ANL2*, a member of the *HD-ZIP* family, was associated with five key downstream genes (*SQE*, *DD*S, *CYP716A53V2*, *β-AS*, *CYP716A52V2*); however, the correlation was relatively weak. *MYB78* exhibited the highest correlation with the *FPS* and *SQE* genes, and *MYB2* with the *CYP716A52V2* gene (Fig. 4C). Transient dual-luciferase (LUC) reporter gene assays confirmed that *MYB78* positively regulates the expression of both *FPS* and *SQE* genes, and *MYB2* positively regulates the expression of the *CYP716A52V2* gene (Fig. 4D, Fig. S15C).

### Spatial distribution of ginsenosides in the three *Panax* root tips

**Figure 5:**
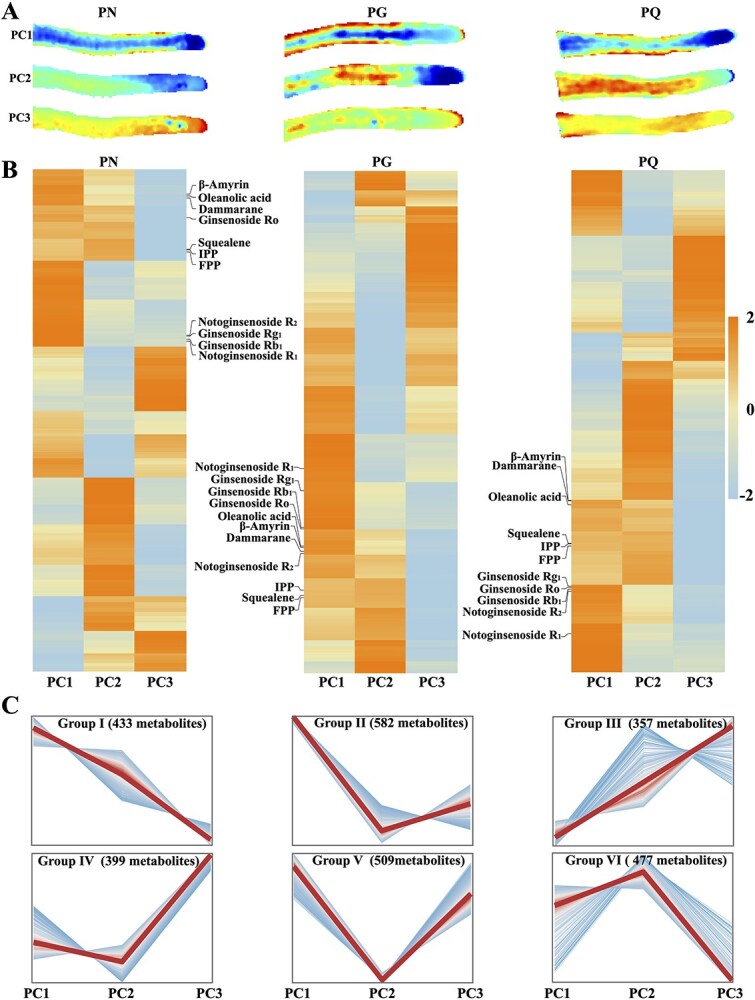
Analysis of MALDI2-MSI datasets from root tips of the three species. (A) Principal component analysis (PCA) of all detected metabolites in root tips of the three species. (B) Heatmap showing relative accumulation levels of each metabolite in different PCs, with ginsenosides and intermediates highlighted. (C) Clustering analysis grouped all metabolites into six distinct clusters.

Spatial distribution of metabolites in the three species' root tips was detected and visualized by MALDI2-MSI (Fig. S17). MSI analysis showed that all metabolites were located on a differentially colored root tip map comprised of 3214 points (a point represents a feature), of which 1807 features were identified as specific compounds (Table S13). The maps separated all features into different locations, which apparently indicated that metabolites accumulated and distributed in tissue-specific patterns (Fig. S17). Principal component analysis (PCA) suggested that all metabolites were separated into three principal components (PCs); PC1, PC2, and PC3 correspond to different known root tip tissues: the apex of root tips (root cap, proliferative cell, and meristematic cell), epidermis (epidermal cell), and vasculature (phloem and xylem), respectively (Fig. 5A).

The heatmap was utilized to visualize the feature intensity of all metabolites across different PCs, with the distribution of ginsenoside and their biosynthetic intermediates highlighted on the heatmap (Fig. 5B). The findings indicated that PG and PQ exhibit more similar metabolite distribution patterns, which may be attributed to their closer genetic relatedness. To determine PC-specific metabolites, clustering analysis classified metabolites into six groups based on their significant contributions to PC separation (absolute loading value ≥0.25 in at least one PC). A total of 457 metabolites were excluded from clustering due to minimal discriminatory power (absolute loading <0.25 across all three PCs) and low biological variability (fold-change <1.5 across all three PCs). These features showed undifferentiated distribution patterns that would introduce noise into the cluster analysis (Fig. 5C). Clustering results showed that Groups I, II, and V included 433, 582, and 509 metabolites, respectively, predominantly accumulated in the epidermis (cell clusters 2/14/17); Groups III and IV contained 357 and 399 metabolites, highly accumulated in the top of root tips corresponding to the root cap (cell clusters 6/7), meristem (cell cluster 0), and proliferative cells (cell clusters 4/5/9/15); Group VI contained 477 metabolites, dominantly distributed in vasculature (cell clusters 3/12/10/13) (Fig. 1B and Fig. 5C, Table S14).

Ginsenosides are the main bioactive ingredients in *Panax* species. A total of 11 related compounds, including ginsenosides and their intermediates, were detected in root tips by MALDI2-MSI analysis. For intermediates, isopentenyl pyrophosphate (IPP), farnesyl pyrophosphate (FPP), and squalene were classified into Group VI, while β-amyrin, oleanolic acid, dammarenediol, ginsenoside Ro/F_3_/Rg_1_/Rg_2_, and notoginsenoside R_2_ were classified into Group I (Table S14). Group VI corresponds to the vasculature and GO functions of DEGs in the vasculature enriched in channel activity, metal ion transport, and lipid transport. This suggested that IPP, FPP, and squalene may be synthesized in the vasculature and then transported to epidermis to provide raw materials for downstream ginsenosides biosynthesis. Group I corresponds to the epidermis. GO enrichment of DEGs in the epidermis focused on isoprenoid and triterpenoid biosynthetic processes, as well as β-AS and UGT activity, which indicated that downstream pathways of ginsenoside biosynthesis may occur in epidermal cells of root tip tissue.

**Figure 6:**
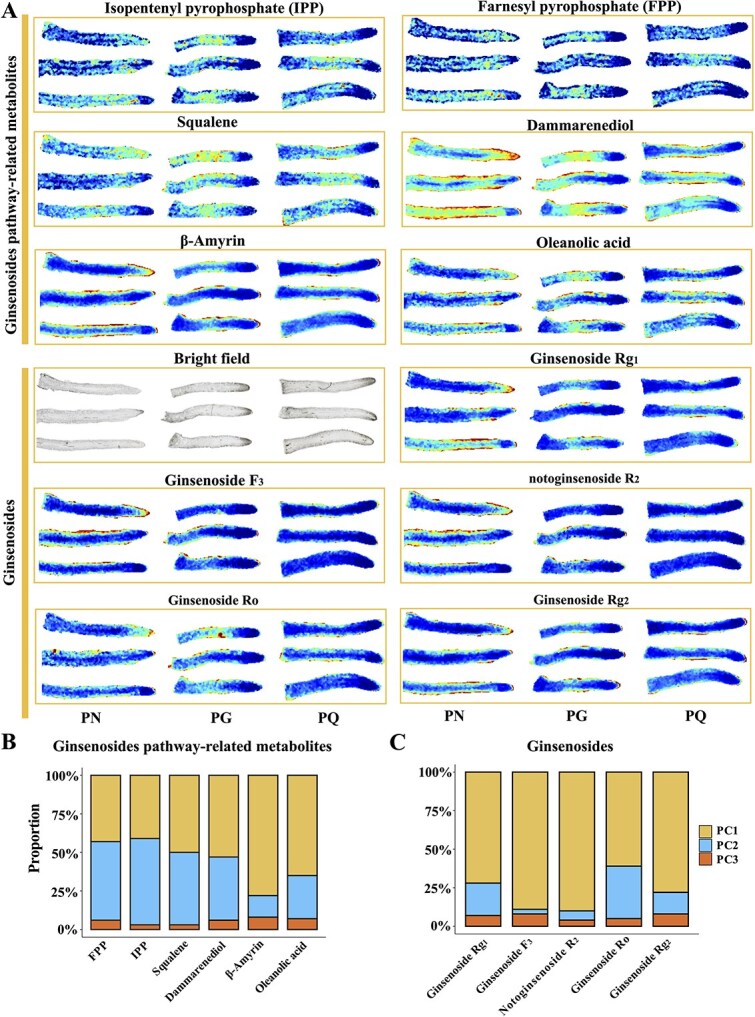
Visualization of ginsenosides and intermediates accumulation in the root tips of three *Panax* species. (A) Tissue-specific distribution patterns of six intermediates and five ginsenosides, as detected by MALDI2-IMS analysis. The color scale ranges from 0% to 100%. (B) Proportions of ginsenosides pathway metabolites across different PCs. (C) Proportions of ginsenosides in different PCs.

MSI analysis directly visualized the *in situ* spatial distribution of ginsenosides and their intermediates. IPP, FPP, and squalene exhibited relatively uniform distribution across the root tip (excluding the apex), while β-amyrin, oleanolic acid, dammarenediol (DDS), ginsenoside Ro/F_3_/Rg_1_/Rg_2_, and notoginsenoside R_2_ showed pronounced accumulation within the epidermis (Fig. 6A). To further validate the distinct spatial patterns observed by MSI, principal component analysis (PCA) was performed on the intensity distribution profiles of all detected metabolites across root tip regions. The analysis revealed that metabolites pre-assigned to Group I (epidermis-enriched: β-amyrin, oleanolic acid, DDS, ginsenosides Ro/F_3_/Rg_1_/Rg_2_, notoginsenoside R_2_) predominantly clustered along the PC1 axis, while metabolites pre-assigned to Group VI (vasculature-enriched: FPP, IPP, squalene) required both PC1 and PC2 for effective separation (Fig. 6B and C). The statistically significant separation of these spatially defined groups along the principal components supports the tissue-specific accumulation patterns of ginsenoside intermediates, as directly visualized in Fig. 6A. Furthermore, the detection of β-Amyrin, oleanolic acid, and ginsenoside Ro in PN root tip by MALDI2-IMS, consistent with LC–MS/MS results, suggests the early synthesis of oleanane-type ginsenosides during root tip development (Fig. 6A).

### Species specificity of ginsenoside accumulation

Widely targeted LC–MS/MS analysis detected 1133 metabolic features (Table S15) following rigorous processing. First, features with >50% missing values (where values below the limit of quantification/LOQ of 0.3 ng/ml, RSD < 15%, were considered missing) across samples were excluded. Second, retained features then underwent KNN imputation (k = 5, Euclidean distance-weighted). These 1133 features can be divided into 20 chemical categories, including amino acids and their derivatives, flavonoids, terpenoids, among others. (Fig. S18A). The contents of oleanolic acid and ginsenoside Ro (oleanane-type ginsenoside) in PN are significantly lower than those in PG and PQ, while the contents of most dammarane-type ginsenosides (such as ginsenosides Rb_1/2/3_, Rg_1/2_, Rc/d/e/f, Rf_2/3_, etc.) in PN are significantly higher than those in PG and PQ (Fig. S18B, Table S16). This result confirmed the analysis based on scRNA-seq and bulk RNA-seq, which showed that the expression levels of key enzyme genes *DDS* and *CYP716A53V2* involved in dammarane-type ginsenosides biosynthesis were significantly higher in PN than in PG and PQ, while the expression levels of key enzyme genes *β-AS* and *CYP716A52V2* involved in oleanane-type ginsenosides biosynthesis were significantly higher in PG and PQ than in PN (Fig. S16). Pseuoginsenoside F11 (ocotillol-type ginsenoside), a unique ginsenoside component in PQ, showed higher contents in PQ root tip than that in PN and PG (Fig. S18B, Table S16).

### The expression patterns of *FAD* gene family and species specificity of unsaturated fatty acids accumulation

A total of 54 *FAD* genes were identified from our scRNA-seq datasets (Table S17). The top five *FAD* genes, ranked by average expression levels, were selected to study their expression distribution across the root tip tissue and pattern among PN, PG, and PQ. Results showed that these genes were specifically overexpressed in the apex of root tip of the three *Panax* species and exhibited higher expression levels in PG (Fig. S19A and B). RNA *in situ* hybridization and RT-qPCR targeting the *FAD2.06* gene confirmed its higher expression in the apex of the root tip, including the root cap, proliferative cells, and meristematic cell populations, and in PG (Fig. S19C–E). TRNs analysis revealed that the *FAD* gene family is widely regulated by *ERF*, *WRKY*, *GATA*, and *C3H* TF families, with *ERF* and *WRKY* families being dominant. Notably, *AT1G16060* in the *ERF* family and *WRKY75* in the *WRKY* family are the two most important regulatory elements, each of which regulates more than half of the genes in the *FAD* families (Fig. S19F). The function of the *FAD* gene family is to synthesize unsaturated fatty acids. In LC–MS/MS analysis results, lipids accounted for 11.12% of the total number of metabolites (Fig. S18A). Among the lipid metabolites, 34 unsaturated fatty acids were identified. The average content of these unsaturated fatty acids in PG was significantly higher compared to that in PN and PQ (Table S18).

## Discussion

The root development and secondary metabolisms are two of the most critical areas of focus in *Panax* species. Root development relies on cell differentiation, which shapes the physical anatomy and specific architecture of the root, enabling it to perform various physiological processes such as material transport and environmental responses [34]. Therefore, dissecting cell differentiation and associated TRNs can provide valuable insights into the functions of specific cell populations and root development. The complexity of secondary metabolism in plant tissues is influenced not only by genetic information but also by the spatial localization of chemicals [30]. Over a hundred ginsenosides have been identified exclusively in *Panax* species, yet their precise sites of biosynthesis within the root tips remain largely uncharacterized [8, 59]. In this study, combining scRNA-seq analysis with spatial metabolomics provides comprehensive data to elucidate developmental transitions and ginsenosides accumulation patterns in *Panax* species root tips.

Plant roots absorb nutrients from the soil environment, traversing radially through all external cell layers (epidermis, cortex, and endodermis), before reaching the central vasculature (Fig. 3A) [34]. The endodermis surrounding the vasculature serves as a critical barrier for selective nutrients absorption, where lignification and suberization construct this physiological checkpoint [6]. Our CCC analysis revealed that the most robust paracrine signaling occurred specifically between the endodermis and cortex (Fig. S12A and B). Key L–R pairs, such as *THE1* and *FER*, were identified as regulators of cell wall integrity and kinase signaling pathways [60]. This finding suggests potential inter-layer communication mechanisms that contributing to the coordinated development and function of the endodermal barrier. Notably, we identified a novel TF, *IAA29*, which directly regulates endodermis suberization. *IAA29*, involved in auxin signal transduction, targets PIF4. Previous studies have demonstrated that Aux/IAA-based auxin signaling modulates root system development, while PIF4 regulates cell elongation in *A. thaliana* [61, 62]. Furthermore, a recent study reported interactions between lignification and suberization, constructing a TF-regulation network for endodermis development in *A. thaliana* roots, suggesting that endodermal suberization may result from both stress responses and developmental regulation [63].

ScRNA-seq analysis revealed that most enzyme genes involved in ginsenoside biosynthesis, particularly downstream genes such as *SQE*, *DDS*, *CYP716A53V2*, *CYP716A52V2*, and *β-AS*, were overexpressed specifically in epidermal cells. This expression pattern may be closely related to the root tip’s response to external stresses, as the epidermis serves as the first barrier against external stimuli. Numerous studies have shown that secondary metabolites produced by plants play a crucial role in responding to adverse environments and are integral to their defense systems for survival [64–66]. Notably, it has been confirmed that *Panax* species produce a large number of specific ginsenosides to combat external pathogen attacks [67]. Overall, the cellular localization of enzyme genes expression involved in ginsenoside biosynthesis provides valuable insights into the biosynthetic locations of bioactive metabolites in *Panax* species. Plant secondary metabolism is largely controlled by complex TRNs, where TFs play irreplaceable roles. Several TF families, including *MYB*, *WRKY*, *bHLH*, and *ERF*, have been shown to regulate ginsenosides biosynthesis, but most studies have focused on different organs of *Panax* species [1, 68, 69]. In this study, we identified highly expressed TFs in epidermal cells and analyzed the TRNs between TFs and enzyme genes involved in ginsenoside biosynthesis, verifying that two key TFs, *MYB2* and *MYB78*, positively regulate ginsenosides biosynthesis. It has been reported that upregulation of *MYB2* promotes anthocyanin accumulation in the leaves of *Dendrobium bigibbum* and aerial stems of *P. notoginseng* [70, 71]. The binary complexes of *MYB78* and *bHLH31* have been proven to regulate ginsenosides accumulation in *P. notoginseng* [72]. These findings will facilitate breeding and genetic improvements of superior *Panax* species.

High-resolution MALDI2-MSI is an exceptional tool for investigating metabolic tissue specificity [73]. In our MSI datasets, the MS features were clustered into the first three PCs, each exhibiting distinct visual distribution characteristics. PC1 demonstrated a predominantly epidermal accumulated pattern, PC2 highlighted vasculature accumulation, and PC3 revealed specific accumulation at the apex (root cap, meristem, and proliferative cells) of the root tip. These results provided comprehensive and precise cellular localization data for ginsenosides accumulation in *Panax* root tip. The biosynthesis of ginsenosides can be divided into upstream and downstream pathways, involving several intermediates [1]. Upstream intermediates, such as IPP, FPP, and squalene, were evenly distributed throughout the root tip except at the apex. This distribution pattern may be attributed to their involvement in the biosynthesis of other triterpenes, including cycloartenol and lanosterol (Figs 4A and 6A). In contrast, downstream intermediates, including β-Amyrin, oleanolic acid, and dammarenediol, which are essential for the biosynthesis of oleanolane- and dammarane-type ginsenosides, predominantly accumulated in the epidermis (Fig. 6A). Additionally, several ginsenosides, such as ginsenoside Rg_1_, Rg_2_, Ro, F_3_, and notoginsenoside R_2_, were enriched in the epidermis, suggesting a potential *in situ* biosynthesis step between downstream intermediates and ginsenosides. The distinct accumulation patterns revealed different biosynthesis sites for various intermediates and highlight potential transport mechanisms of secondary metabolites between distinct cell types.

The *FAD* gene family, an environmentally adaptive gene family, encodes enzymes that synthesize unsaturated fatty acids. 85 *FAD* genes were reported in the PG genome, more than three times that in the model plants *A. thaliana*, *O. sativa*, and *Solanum lycopersicum* [74]. The expansion of *FAD* gene family might be closely related to the long-term survival of PG in low temperatures. Regarding the three species, PN mainly grows in warm freeze-free areas with high altitudes in southwest China, PQ originated from North America and has been broadly cultivated in China, and PG is widely distributed in Northeast Asia where freezing temperatures prevail or are even lower in the winter [74]. In this study, expression level of *FAD* gene family in PG was higher than that in PN and PQ, and LC–MS/MS analysis showed the highest contents of unsaturated fatty acids in PG, suggesting that the *FAD* gene family plays a significant role in the long-term adaptation of PG to cold environments (Fig. S19A and E, Table S17). Additionally, the *FAD* gene family was specifically overexpressed in proliferative, meristematic, and root cap cell populations, indicating that the apex of the root tips is most responsive to cold stress. TRNs analysis revealed that *AT1G16060* and *WRKY75* were the two most crucial positive regulatory elements of *FAD* gene family expression. *AT1G16060*, a positive regulator of the abscisic acid (ABA) response, regulates seedling growth. *WRKY75* participates in regulating ABA-mediated seed germination in *A. thaliana* [75]. Numerous studies have indicated that increased ABA biosynthesis can enhance plant tolerance to cold stress. Therefore, it can be inferred that *AT1G16060* and *WRKY75* might regulate *FAD* gene family expression and ABA biosynthesis-related genes simultaneously to improve cold tolerance in *Panax* species. These results will contribute to the breeding of freeze-tolerant varieties of other *Panax* species.

## Conclusion

This study presented a single-cell transcriptome and spatial metabolome profile of *Panax* species. We conducted a comprehensive analysis of the developmental trajectory of the endodermis, cell type-specific expression patterns of key enzyme genes involved in ginsenoside biosynthesis, cell–cell communication analysis, and the expression profiles of the *FAD* gene family. Additionally, we dissected TRNs involved in these critical biological processes at the cellular level. Through *in situ* hybridization, RT-qPCR, tissue staining, transient dual-LUC reporter assays, and EMSA, we identified key TFs, including *IAA29* (which directly regulates endodermis suberization), *MYB2* and *MYB78* (that positively regulate ginsenoside biosynthesis), and *WRKY75* and *AT1G16060* (which mediate the expression of the *FAD* gene family). Collectively, these findings will facilitate the selection of elite *Panax* cultivars through molecular breeding and provide novel insights into the transcriptional regulatory mechanisms governing secondary metabolism in medicinal plants.

## Materials and methods

### Protoplast preparation, scRNA-seq library construction and sequencing

Mature seeds of PN, PG, and PQ were sterilized and stored in moist sands at approximately 15°C with 70%–75% relative humidity to promote germination. After germination, root tips (~5 mm in length from the apex of the root tip) were collected (50 root tips per species pooled). Single-cell suspensions were prepared as follows: root tips were cut into thin slices (~0.1 mm thickness) and immediately digested in RNase-free enzyme solution (20 mM KCl, 20 mM MES, 10 mM CaCl_2_, 5 mM β-mercaptoethanol, 1.5% Cellulase R10, 0.5% Mecerozyme R10, 0.4 M mannitol, 0.1% BSA) in the dark at room temperature for 3 hours. After digestion, protoplasts were filtered through cell strainers (40 μm diameter) three times, centrifuged at 300 × *g*, and washed three times using WI solution (20 mM KCl, 4 mM MES, 0.5 M mannitol). Protoplast viability was assessed using a trypan blue exclusion assay, with all three samples demonstrating ≥80% cell viability, and dead cells were removed using a Miltenyi Dead Cell Removal Kit. Subsequently, the concentration was adjusted to approximately 1500 cells/μl. The single-cell suspension for each species was loaded onto a 10x Chromium Single Cell 3′ Platform (10x Genomics, Pleasanton, CA) to generate respective single-cell GEMs. ScRNA-seq libraries were constructed using the Chromium Single cell 3′ GEM and library reagent kit v3 (10x Genomics, Pleasanton, CA) according to the user manual. The quality of the cDNA libraries was evaluated using an Agilent 2100 Bioanalyzer. Libraries were sequenced on an Illumina NovaSeq 6000 sequencer (San Diego, CA 92121, USA) to obtain 150 bp paired-end reads.

### Pre-processing of raw scRNA-seq data

The reference genome was downloaded from NCBI BioProject number PRJNA658419. Raw scRNA-seq data in SRA format were converted to fastq format using fastq-dump v2.8.0 and then imported into Cell Ranger pipelines v4.0 (10x Genomics) for quality control, barcodes generation, features extraction, and matrix construction. After filtering empty GEMs, the reference index was constructed using the ‘cellranger mkref’ function, and cell-gene expression counts were generated with the ‘cellranger count’ function for subsequent analysis.

### Data filtering and doublet detection

The barcodes, features, and matrix files were converted into a gene-cell matrix in R using Seurat package v3.3.0 [76]. To maximize effective information while removing low-expression genes and low-quality cells, we filtered out genes expressed in fewer than three cells and retained only cells expressing more than 200 genes and 500 counts, with mitochondrial gene expression below 10%. Protoplast-specific genes were identified based on their similarity to *Arabidopsis* genes [77]. The proportion of protoplast-specific genes in each cell cluster was calculated using the ‘Percentage FeatureSet’ function to assess their impact on cell clustering. Doublets were identified using the DoubletFinder package v2.1.0 [78]. The ‘paramSweep_v3’ function (with PCs set to 1:50) was used to determine optimal parameter nExp and pK. For PN, PG, and PQ, the parameters were set as follows: nExp = 265, pN = 0.25, pK = 0.29; nExp = 396, pN = 0.25, pK = 0.28; nExp = 1198, pN = 0.25, pK = 0.11, respectively. Only cells identified as ‘singlets’ were retained for downstream analysis. After doublets filtration, 5761, 7026, and 11 638 cells from the PN, PG, and PQ samples, respectively, were included in the cell-gene expression matrix for further analysis.

### Data integration, cell clustering, and marker genes identification

The normalized cell-genes expression matrices from PN, PG, and PQ were integrated into a single object using the Harmony v0.1.1 package [43]. The ‘CellCycleScoring’ function was applied to calculate cell cycle score for each cell type, estimating the influence of cell cycle genes on clustering. Gene expression levels were normalized using the ‘NormalizeData’ function with default parameters and scaled using the ‘ScaleData’ function. We identified the top 2000 highly variable genes using the ‘FindVariableFeatures’ function (selection.method = ‘vst’) and performed linear PCA (principal component analysis) dimensional reduction using the ‘RunPCA’ function (npcs = 50). Based on the ‘ElbowPlot’ results, we selected the appropriate PCA dimensions for non-linear dimensional reduction and visualization using the ‘RunTSNE’ and ‘RunUMAP’ functions. To determine optimal clustering resolution, we used the clustree R package (v0.5.1) to systematically evaluate cluster stability across a resolution parameter range (0.1–1.2). Resolution = 0.6 was ultimately selected based on minimal over-clustering artifacts and the preservation of cell type marker genes. Cell were clustered using the ‘FindNeighbors’ (dims = 1:50) and ‘FindClusters’ (resolution = 0.6) functions. Cluster-specific marker genes were identified using the Wilcoxon rank-sum test via Seurat’s FindAllMarkers function, with Benjamini-Hochberg correction applied for multiple hypothesis testing. Differential expression thresholds were defined based on biological relevance: genes with a log2(fold change) ≥0.25 and adjusted *p* < 0.05 were categorized as differentially expressed, whereas those with higher specificity (log2(fold change) ≥ 0.58 at the same significance level, adjusted *p* < 0.05) were classified as cluster-enriched markers.

### Pseudo-time analysis of cell differentiation

We used the Monocle2 package v2.20.0 [79] to infer the differentiation trajectory of specific cell types. Target cell types were extracted, and genes expression variance across cells was computed using the ‘dispersionTable’ function. Variable genes were used to define a differentiation progress, and the ‘DDRTree’ method reduce dataset dimensionality to two components. The developmental trajectory was inferred using the ‘orderCell’ function, and the pseudo-time trajectory was plotted using the ‘plot_cell_trajectory’ function. Pseudo-time-dependent and branch-dependent genes were determined using the ‘BEAM’ function and visualized with the ‘plot_genes_branched_heatmap’ function. Dynamic expression changes in branch-dependent genes along the pseudo-time trajectory were visualized using the ‘plot_pseudotime_heatmap’ function.

### RNA velocity analysis

RNA velocity was analyzed using velocyto.py v0.18 and the scVelo v0.2.5 package [80, 81]. Spliced (emat) and unspliced (nmat) transcripts were counted using the Velocyto. py package. Three loom files from PN, PG, and PQ, generated by Cell Ranger, were combined using ‘loompy’. The merged loom file was imported into scVelo, and cell subpopulations of interest were extracted. RNA velocity values for each gene in each cell were calculated and embedded into a low-dimensional space. Finally, UMAP embeddings were exported to Seurat for visualization of individual cell RNA velocity.

### Enrichment and clustering analysis

GO and KEGG enrichment analysis of cluster-enriched and branch-dependent genes were performed using the ClusterProfiler package [82]. All genes were used as background gene sets for the enrichment analysis. Additionally, we conducted clustering analysis on all genes using the Mfuzz package v2.52.0 [83] to elucidate the correlation between dynamic gene expression patterns and biological functions.

### TFs regulatory network analysis

In total of 2674 TFs were identified from the PN reference genome based on alignment results with iTAK and PlantTFDB v5.0 [84, 85]. These TFs were classified into 88 different families, with *MYB*, *MYB*-related, *bHLH*, *bZIP*, *ERF*, *NAC*, *WRKY*, and *C2H2* containing more members than other families (Table S11). To identify novel and important TFs involved in ginsenoside biosynthesis, cell differentiation, and environmental adaptation in *Panax* species, we inferred TRNs between TFs and key enzyme genes associated with these critical biological processes, following methods described in relevant literatures [46, 69].

### Cell–cell communication analysis based on ligand-receptor interactions

We performed CCC analysis to predict L–R pairs interactions in PN, PG, and PQ using the PlantPhoneDB database and its corresponding R package v1.0.0 [5]. L–R pair predictions were based on homologous alignment. To identify cell type preferences, we computed the ratio of observed to expected (Ro/e) for all cell types using the chi-square test. Significant L–R pairs between pairwise cell types were then identified using the average scoring approach (pairs exceeding a predefined significance threshold based on the average score).

### Bulk RNA-seq and analysis

Root tips were collected as described above and immediately frozen in liquid nitrogen for bulk RNA sequencing. Total RNA from each sample was extracted, and libraries were constructed and sequenced on the Illumina NovaSeq 6000 platform (San Diego, CA, USA) to generate 150 bp paired-end reads. Adapter sequences, ploy-N regions, and low-quality reads were removed using Trimmomatic v0.39 [86] to obtain clean reads. The clean reads were then aligned to the PN reference genome using STAR v2.7.2 [68, 87]. Gene expression levels were calculated and normalized using featureCounts v2.0.1 [88] and the Python package rnanorm v1.4.0. Finally, Pearson correlations between gene expression levels from scRNA-seq and bulk RNA-seq datasets were computed using R.

### Sample preparation and data processing for spatial metabolome

Fresh root tips (~5 mm) were embedded in 10% croscarmellose sodium (Sigma, Germany) on dry ice. Tissues were cut into 20 μm thick slices using a Leica CM1950 freezing microtome. The slices were transferred onto MALDI2 special conductive glass slides and dried in a vacuum dryer for 30 minutes. A MALDI2 special matrix solution was evenly sprayed on the glass slides containing root tip slices using a TM-Sprayer matrix sprayer. The conductive glass slides were placed on the target plate of timsTOF flex MALDI2 instrument, and then detection area (imaging resolution = 50 μm) was selected using Bruker data imagine software. Scanning the glass slide, ionized molecules released from the target site were detected by MS, generating raw MSI data files.

The raw MSI files were important into SCiLS™ Lab v2021 software (Bruker Daltonics) for preprocessing, including baseline smoothing, peak alignment, and data normalization. Unsupervised spatial clustering was applied to the imaging pixels of the target area using the method provided by SCiLS software. Regions with similar metabolite distribution patterns were colored identically to visually define different regions of the slices at the molecular level. Metabolite annotation and qualitative analysis were performed using a Bruker Metaboscape™ workstation. Specifically, metabolites were identified by searching against the Bruker MetaboBase Library 3.0 (with a molecular mass error < 10 ppm). The distribution pattern of all identified metabolites in root tip was visualized using the Mfuzz clustering algorithm.

### Sample preparation and data processing for LC–MS/MS

Fresh root tips (~5 mm) from PN, PG, and PQ were ground into powder in liquid nitrogen, respectively. Three biological replicates were prepared for each species. For each sample, 100 mg of powder was dissolved in 500 μl of 80% methanol (Thermo Fisher, USA). The solution was vortexed for 3 minutes, incubated in an ice bath for 5 minutes, and centrifuged at 15 000 *g* for 20 minutes at 4°C using a cryogenic centrifuge (scilogex, USA). A certain amount of supernatant was diluted with MS-grade water to achieve a final methanol content of 53%, and then centrifuge again under the same conditions. The resulting supernatant (10 μl/sample) was used for LC–MS/MS detection. The gradient elution conditions for UPLC (Exion LC with Xselect HSS T3 chromatographic column) are shown in Table S19.

### Microstructure observation of seeds and root tips

Germinating seeds and root tips (~5 mm) of PN, PG, and PQ were fixed in FAA fluid for 24 hours, then embedded in paraffin and sectioned for conducting HE staining (seeds) and safranin O green staining (root tips). The anatomical structure of seeds and root tip were observed using microscope.

Root tips (~5 mm) of PN, PG, and PQ were fixed in Carnoy’s fluid for 24 h, followed by paraffin embedding and sectioning for casparian strip and suberin staining. For casparian strip staining, cross-sections (4 μm thick) were cut from the region 2 ~ 3 mm from the apex of root tip and observed under fluorescence microscopy using a DAPI filter after staining. For suberin staining, cross-section was cut from the region 4 ~ 5 mm from the apex of root tip and observed under fluorescence microscopy using a GFP filter.

### RNA *in situ* hybridization

Probe sequences for target genes (antisense) and their corresponding negative control sense sequences are listed in Table S20. Root tips were fixed in FAA solution (formalin:acetic acid:70% ethanol = 1:1:18) for 24 hours at 4°C. After paraffin embedding, sections (8 μm thickness) were prepared using a Leica RM2235 microtome. For experimental groups: sections were dewaxed in xylene, rehydrated through an ethanol gradient, and digested with 10 μg/ml proteinase K at 37°C for 20 minutes. Following pre-hybridization (2 hours at 42°C in hybridization buffer), antisense DIG-labeled probes (200 ng/ml) were applied and hybridized overnight at 42°C. For negative controls, parallel sections underwent identical processing but were hybridized with equimolar sense probes under the same conditions. After hybridization, all slides were stringently washed (2× SSC/0.1% SDS at 42°C, 0.1× SSC/0.1% SDS at 37°C). Non-specific binding was blocked with 1% BSA for 1 hour before incubation with anti-DIG-AP antibody (1:500 dilution; Jackson ImmunoResearch) for 2 hours at room temperature. Chromogenic detection was performed using NBT/BCIP substrate (BOSTER Biological Technology) for 4–12 hours in the dark. The reaction was terminated with TE buffer (10 mM Tris–HCl, 1 mM EDTA, pH 8.0). Critical validation: Sense-probe controls consistently exhibited no specific staining, confirming probe specificity. Slides were counterstained with 0.1% Fast Red, mounted in Permount, and imaged using an Olympus BX53 microscope.

### RT-qPCR verification

PN, PG and PQ root tips (~5 mm) were divided into two sections: a 1-mm segment from the apex of root tip and the remaining 4 mm. 6 samples were collected in total, with 3 biological replicates per sample, each replicate consisting of 50 root tips from corresponding seeds. Total RNA was extracted using Trizol Kit (Thermo Fisher Scientific, MA, USA), and cDNA was synthesized using SynScript^®^III RT SuperMix Kit (TsingkeBiotech, Beijing, China). Specific primer sequences for the *FAD2.06* gene were designed using Primer-BLAST (https://www.ncbi.nlm.nih.gov/tools/primer/blast/) for RT-qPCR amplification. The expression level of the target gene was normalized to the *β-actin* internal reference gene. The specific primer sequences for *FAD2.06* and *β-actin* gene are listed in Table S21.

### Transient dual-LUC reporter assay

The promoter sequences of *COMT*, *FPS*, *SQE*, and *CYP716A52V2* genes were cloned into pGreenII0800-LUC vectors, respectively. Subsequently, the following constructs were co-transfected into *Agrobacterium* CV3101: *COMT* with pGreenII 62-SK-*IAA29*, *FPS* and *SQE* with pGreenII 62-SK-*MYB78*, and *CYP716A52V2* with pGreenII 62-SK-*MYB2*. *Agrobacterium* suspensions were infiltrated into 4-week-old tobacco leaves using a 1 ml syringe. Tobacco plants were incubated under low light for 1 day and then under normal light for 2 days. Tobacco leaves were collected and imaged using the Tanon-5200 chemiluminescence imaging system (Tanon Science and Technology). Firefly and Renilla luciferase activities were quantified using a dual-luciferase assay kit (Yeasen Biotech, Shanghai, China) according to the manufacturer’s instructions. The LUC expression level was normalized by calculating the ratio of LUC to REN. Promoter sequences of the genes and CDS sequences of TFs are listed in Table S22.

### Electrophoretic mobility shift assay

The 2000-bp upstream promoter sequence of the *COMT* gene was extracted from *P. notoginseng* genome [69] and analyzed via PlantCARE website (http://bioinformatics. psb.ugent.be/webtools/plantcare/html/) to identify *cis*-element. The cDNA sequence of *IAA29* was cloned into pET-28a vector for his-tag fusion. The recombinant protein was expressed in *Escherichia coli* and purified using His60 Ni Superflow. Protein purity was verified by 12% SDS-PAGE. EMSA was performed using the Light Shift Chemiluminescent EMSA kit (Beyotime). Biotin-labeled probes containing the untypical AuxRE binding element (TGTCCC) were designed from the promoter. Unlabeled probes served as competitors, probes with mutated AuxRE cores (TGTCCC→GGGGGG) were negative controls. The EMSA probe sequences are provided in Table S23.

## Supplementary Material

Web_Material_uhaf202

## Data Availability

All raw sequencing dataset involved in this paper have been submitted to NCBI under accession number PRJNA1046239 and PRJNA1045109.
