# Supplementary material for: Integrated single-cell transcriptomics and spatial metabolomics unveil cellular differentiation and ginsenosides biosynthesis in Panax root tips
Source: Hortic Res. 2025 Jul 31;12(11):uhaf202. doi: 10.1093/hr/uhaf202 (PMC12574545; doi:10.1093/hr/uhaf202)
Supplement: Web_Material_uhaf202 [file web_material_uhaf202.zip › Supplementary Figure.docx]

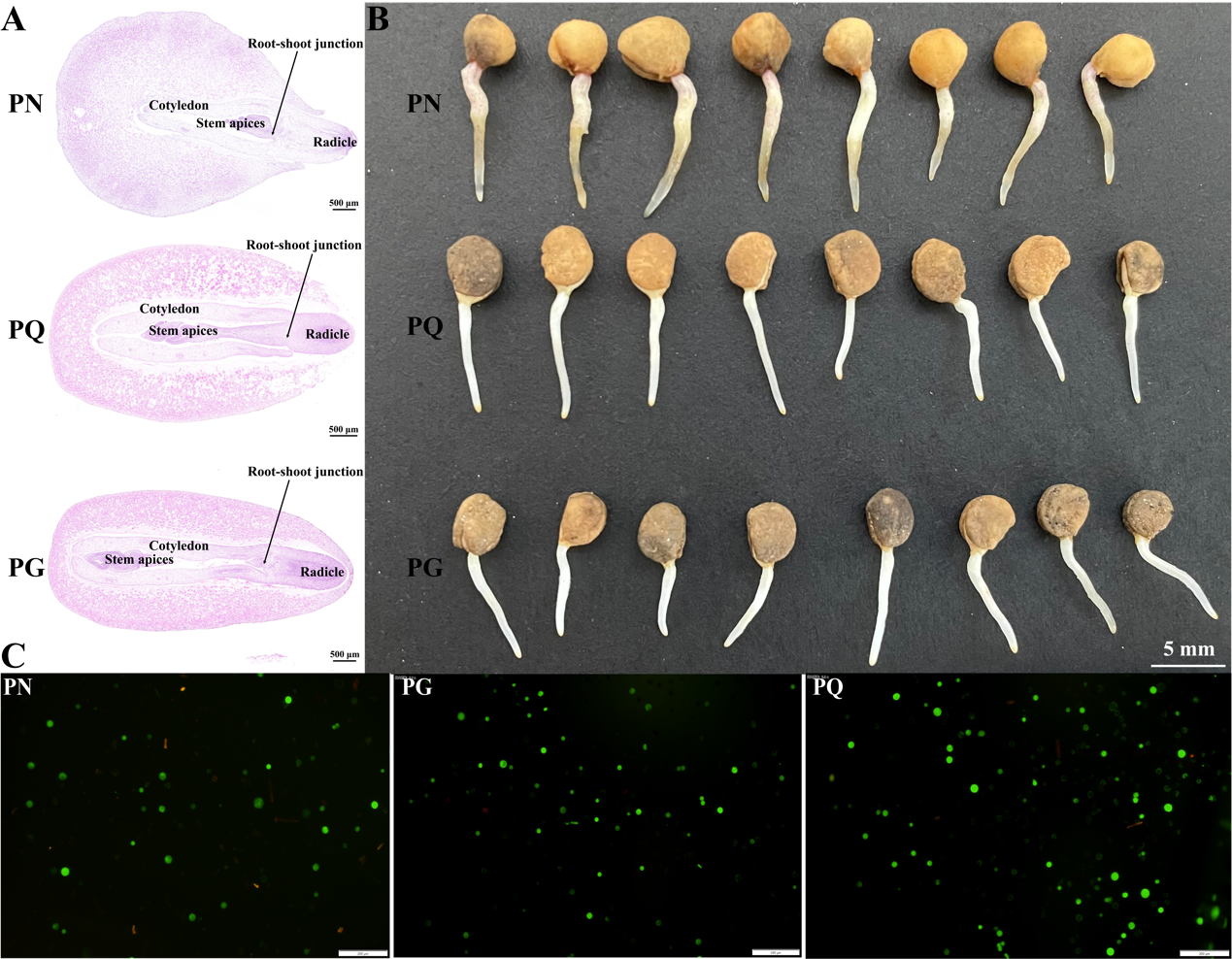


**Figure S1.** Morphological characteristics of germinating seeds and root tip, as well as protoplasts of PN, PG, and PQ. **A** Germinating seeds of PN, PG, and PQ. **B** Root tip morphology of PN, PG, and PQ. **C** Protoplasts of PN, PG, and PQ root tips.


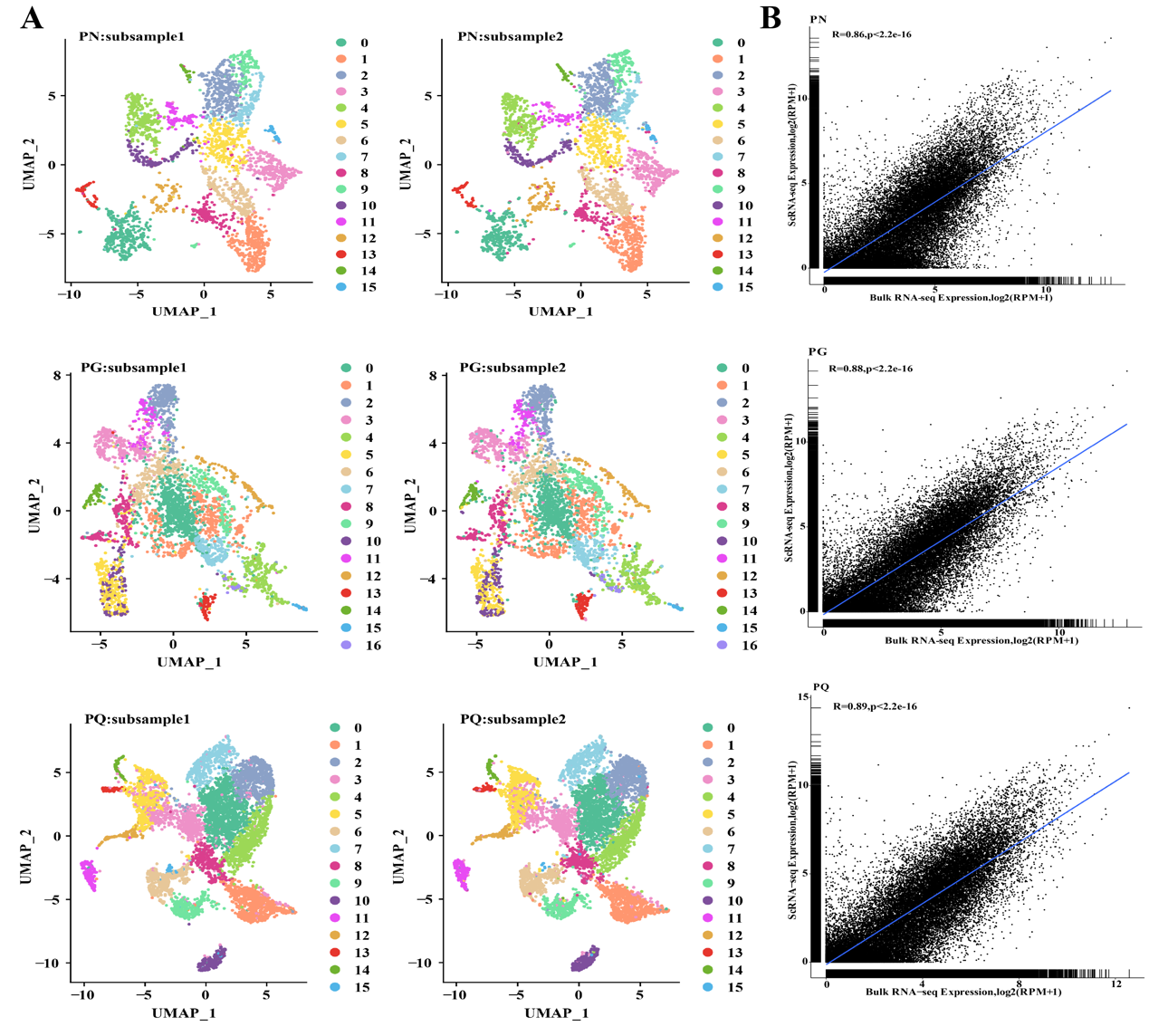


**Figure S2.** Robustness evaluation of scRNA-seq results. **A** UMAP visualization of two randomly selected subsamples from PN, PG, and PQ. **B** Pearson correlation analysis between scRNA-seq datasets and bulk RNA-seq datasets for PN, PG, and PQ. R presents Pearson’s correlation coefficient.


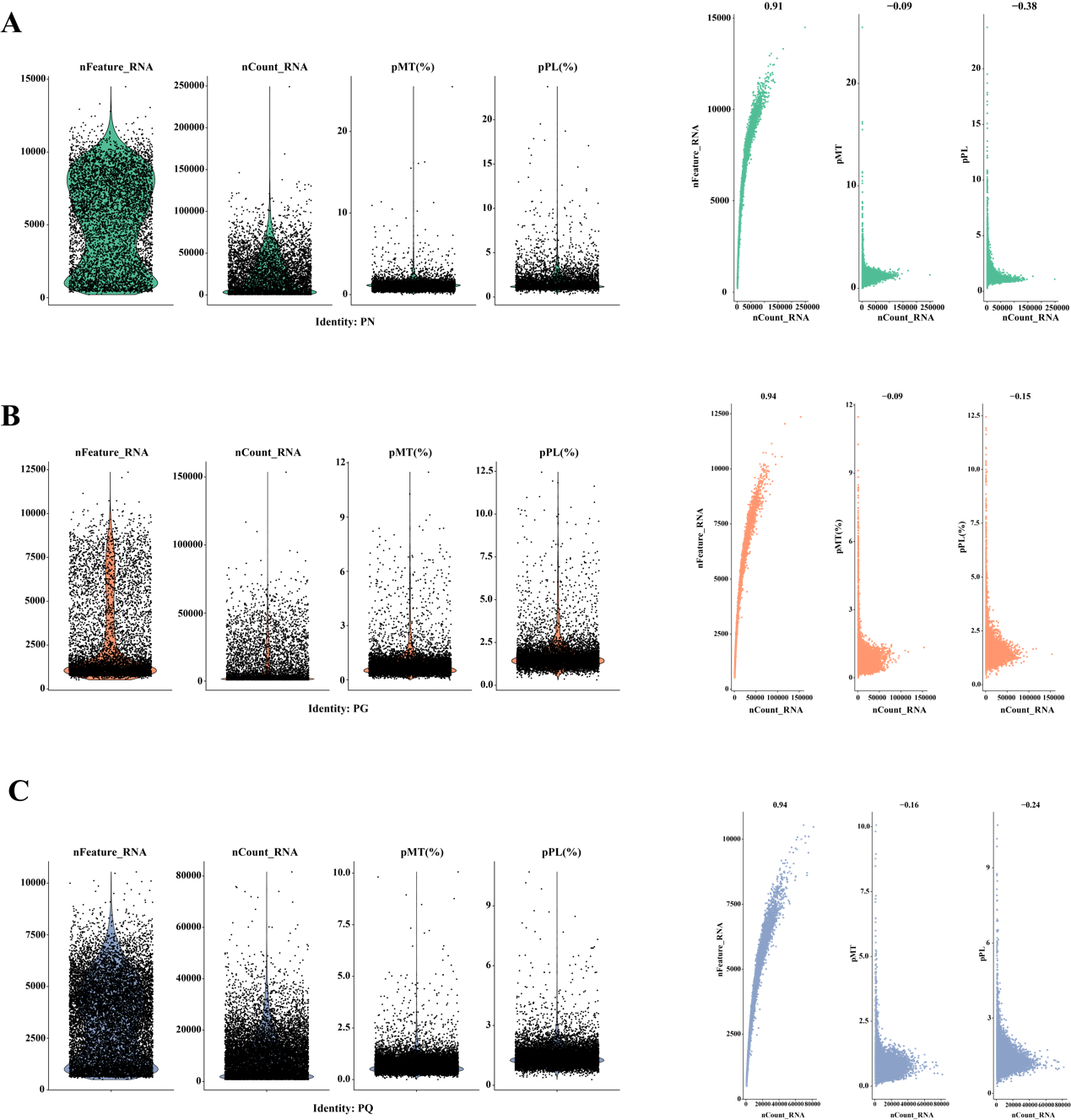


**Figure S3.** Quality control results for scRNA-seq dataset of PN(**A**), PG (**B**), and PQ (**C**).


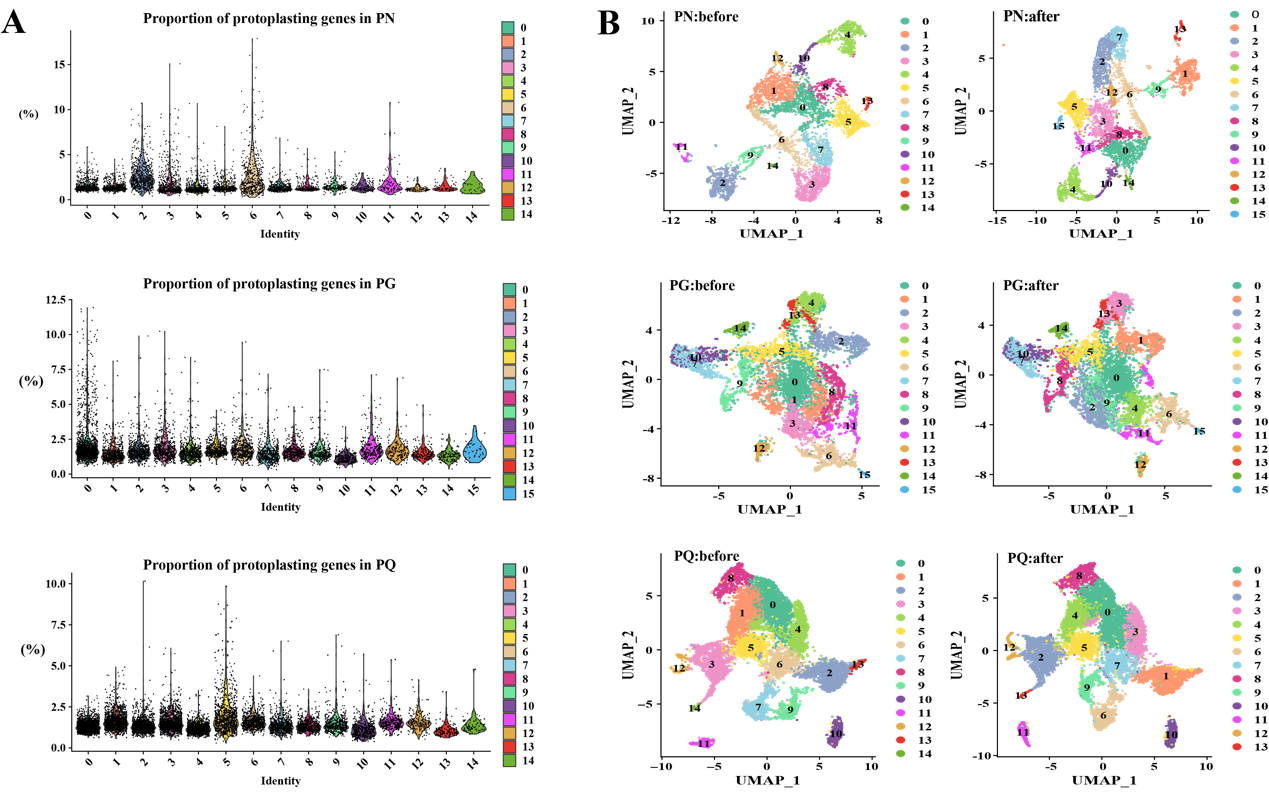


**Figure S4.** Evaluation of the influence of protoplasting genes on cell clustering. **A** Expression proportions of protoplasting genes in each cell cluster of PN, PG, and PQ. **B** UMAP visualization of cell clustering for PN, PG, and PQ before and after excluding the influence of protoplasting genes.


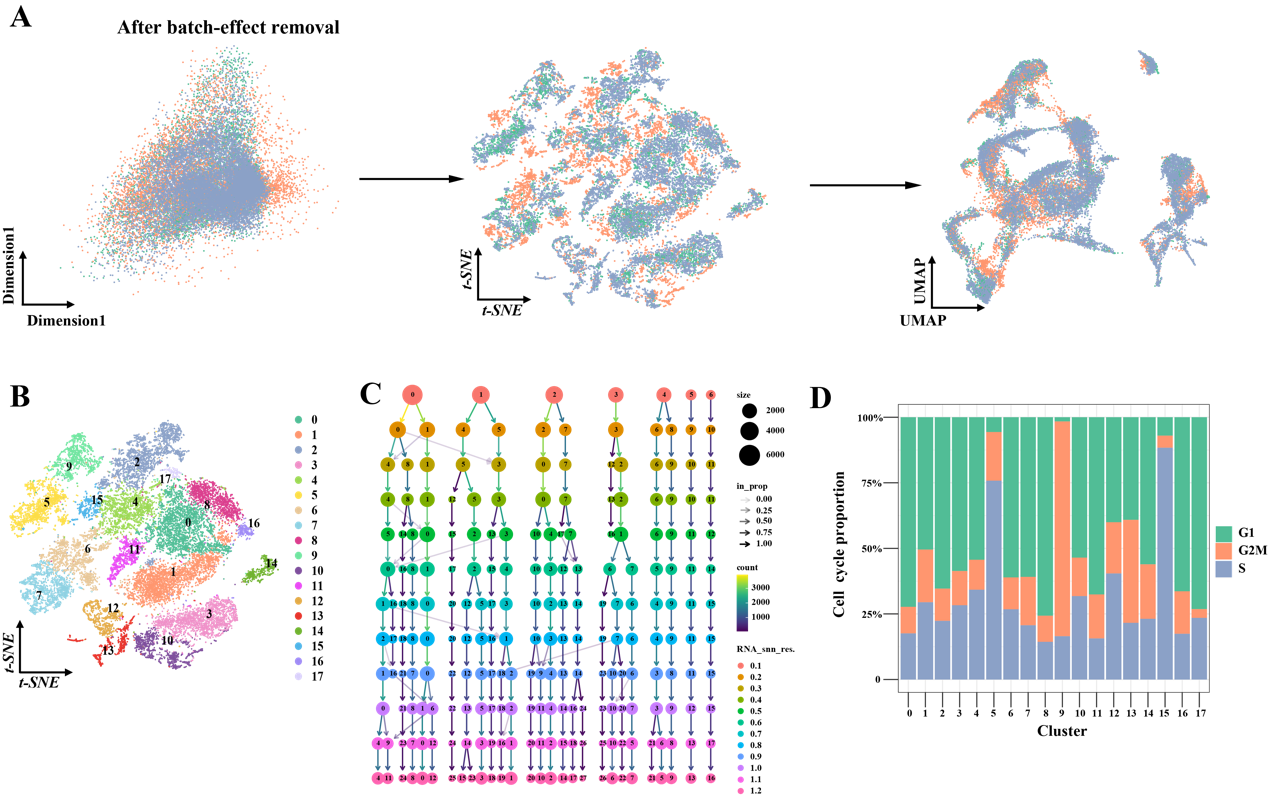


**Figure S5.** Correction of batch effects and the percentage of cell cycle phases. **A** Scatterplot showing the top two dimensions. Batch-effect among the three samples were corrected using the Harmony algorithm. **B** *t*-SNE visualization of the three samples after Harmony correction. **C** The testing results of clustree clustering with resolution from 0.1 to 1.2. **D** Percentage of cells in G1, S and G2/M phases within each cell cluster.


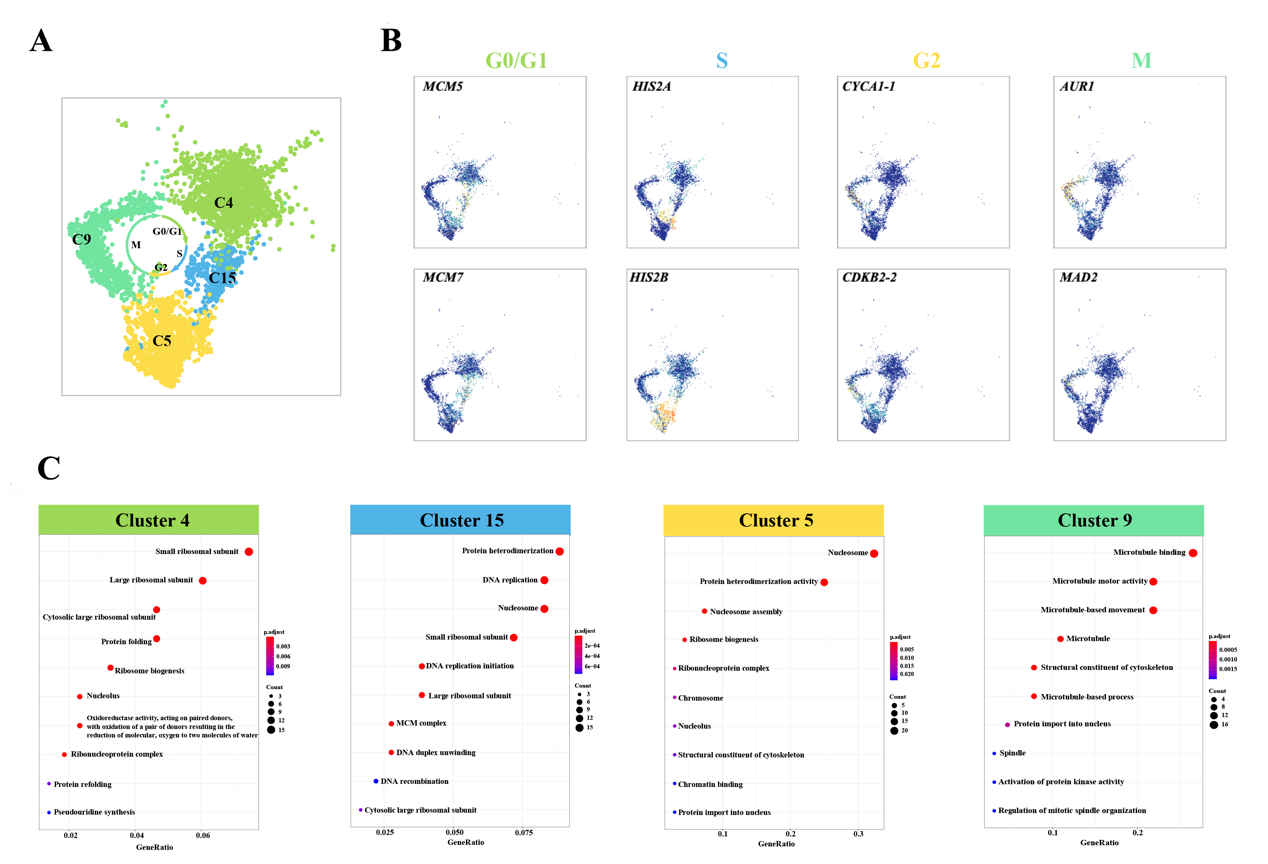


**Figure S6.** Expression profile of cell cycle genes in proliferative cells and GO enrichment results for corresponding cell clusters. **A** UMAP visualization of the proliferative cell population. **B** Expression patterns of cell-cycle genes in cluster 4, 15, 5, and 9. **C** GO enrichment analysis results for cluster 4, 15, 5, and 9.


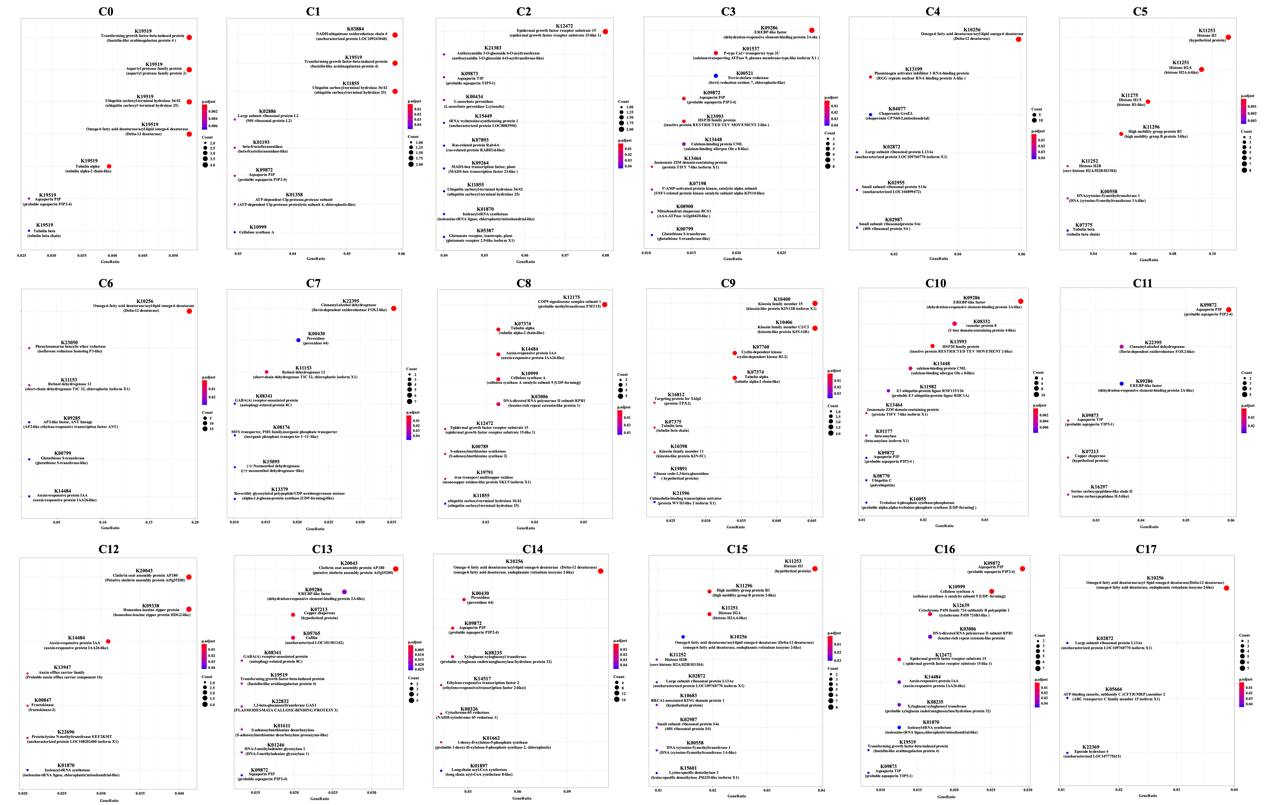


**Figure S7.** KEGG pathway enrichment results for differentiated expressed genes (DEGs) in each cell cluster. Scatter plot displaying KEGG pathway enrichment results for DEGs in each cell cluster. Only pathways with an adjusted *p*-value ≤ 0.05 are shown.


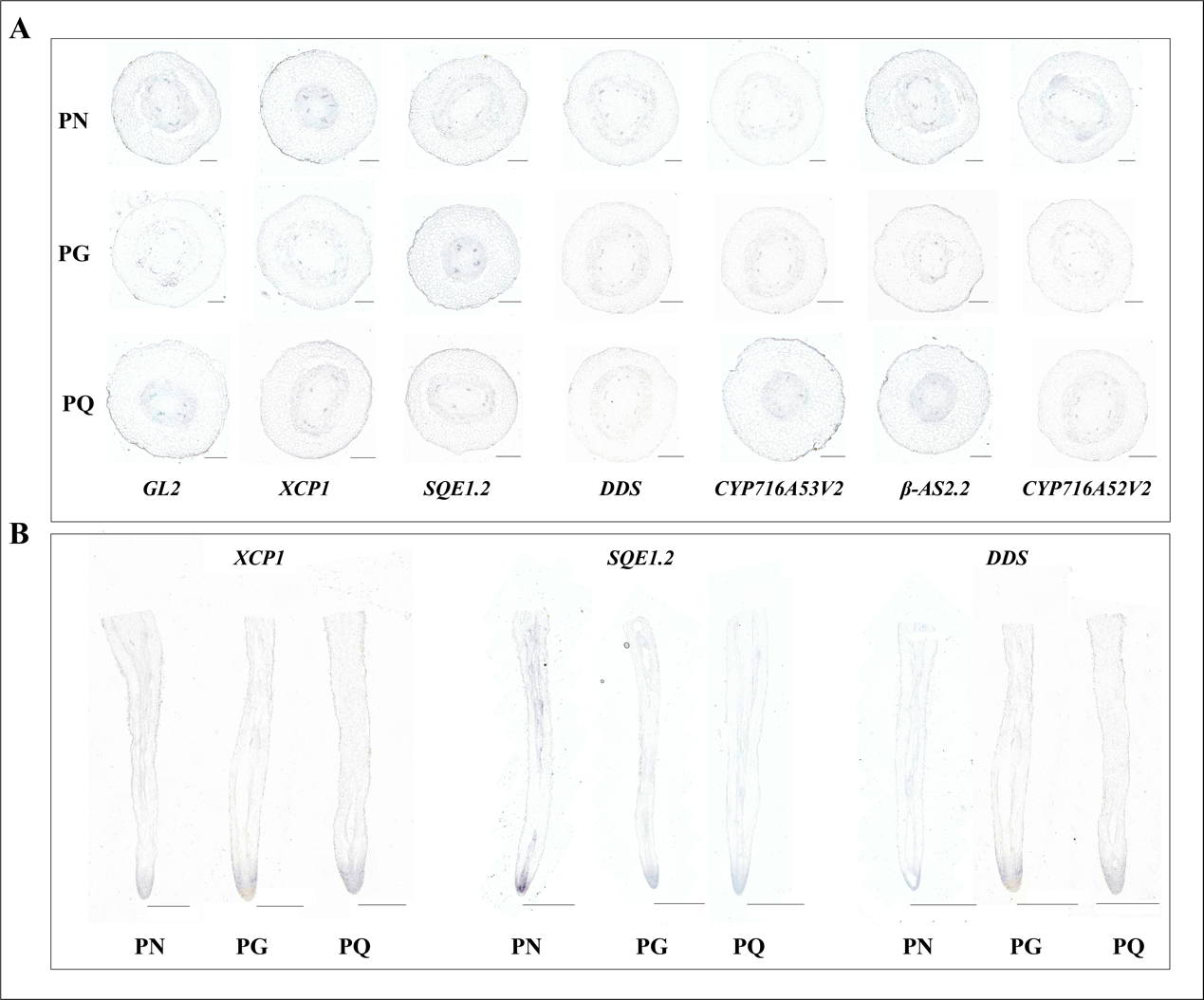


**Figure S8.** Control experiments for RNA *in situ* hybridization. **A** Cross-sectional view of control RNA *in situ* hybridization, scale bars represent 200 μm*.* **B** Longitudinal-sectional view of control RNA *in situ* hybridization, scale bars represent 1000 μm.


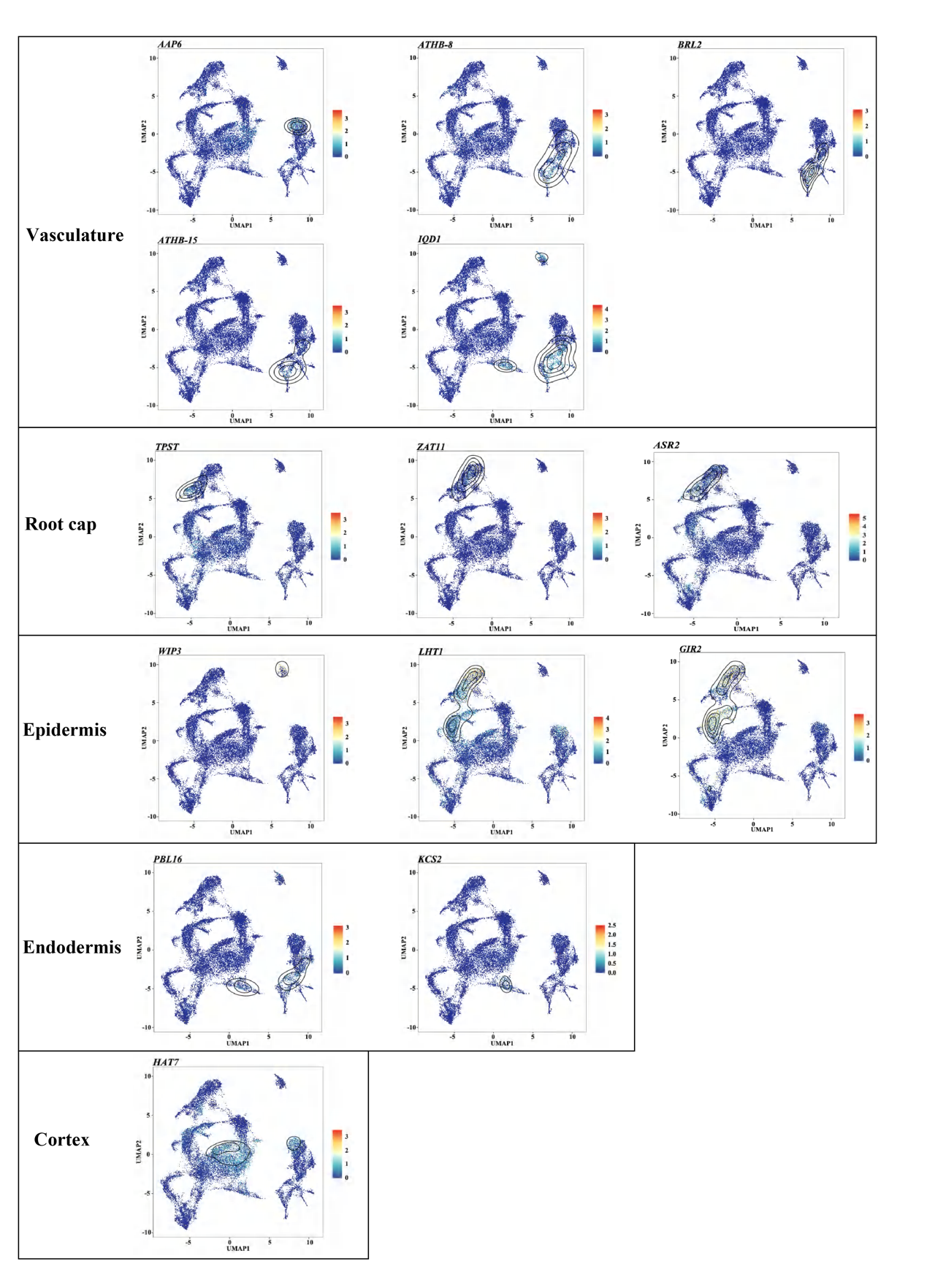


**Figure S9.** Identification of cell-type marker genes in *Panax* species. Featureplot showing the expression patterns of several genes identified as cell-type marker genes in *Panax* species.


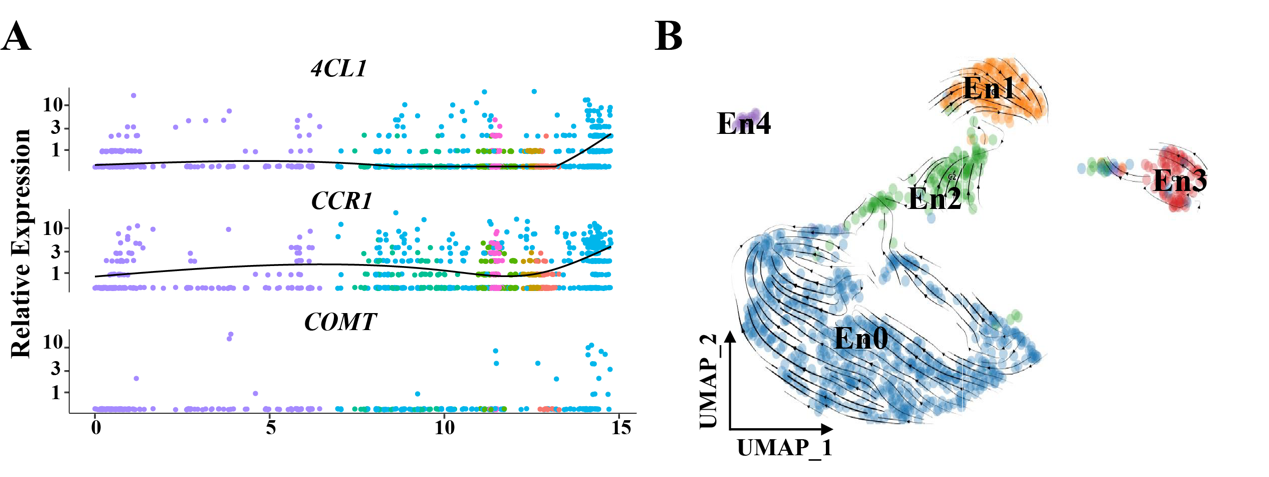


**Figure S10.** Expression dynamics of three key genes involved in the late-stage endodermis differentiation (**A**) and RNA velocity field of sub-cell clusters of the endodermal cell population projected onto a UMAP plot based on scVelo. Arrows represent direction and average RNA velocity (**B**).


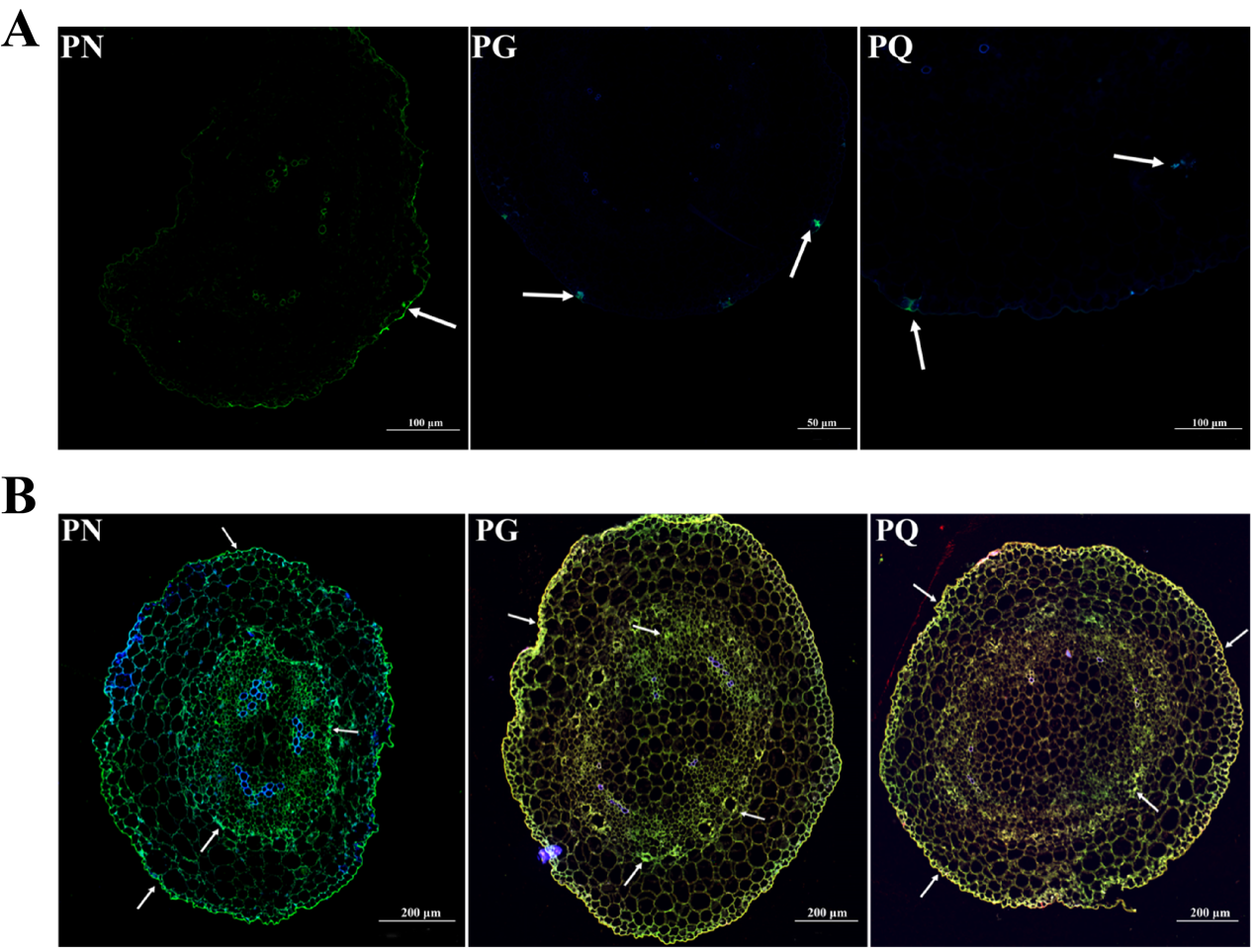


**Figure S11.** Staining results for casparian strip and suberin in root tips of PN, PG, and PQ. **A** Staining results for the casparian strip in root tips of PN, PG, and PQ. **B** Staining results for suberin in root tips of PN, PG, and PQ.


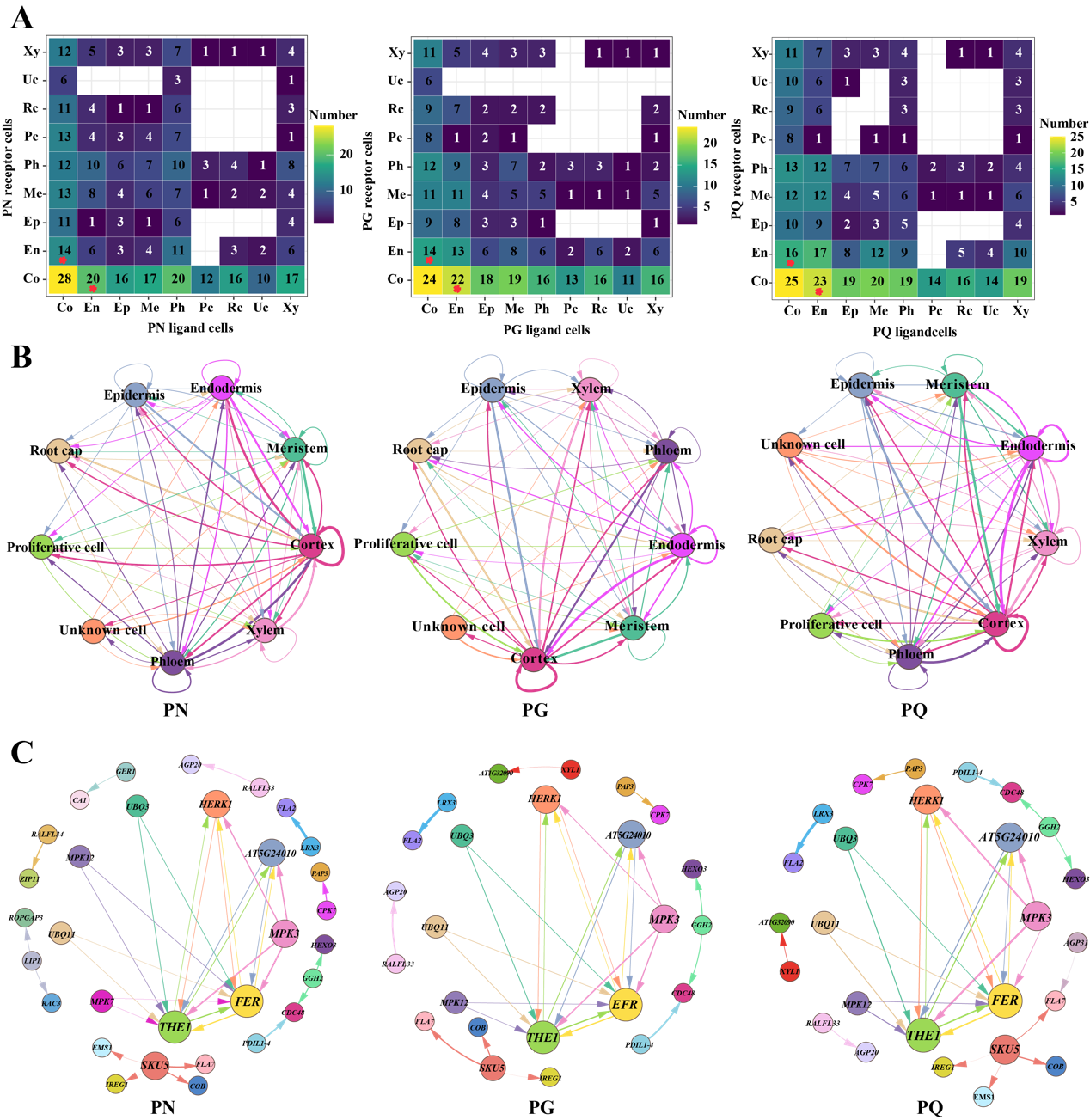


**Figure S12.** Identification of cell-cell communication and ligand-receptor interactions. **A** The number of ligand-receptor pairs between pairwise cell types in PN, PG, and PQ. Row represents cell that express ligand and column represents cell that express receptor. Blank indicates that no corresponding ligand-receptor pairs have been identified. * highlights the number of ligand-receptors between endodermis and cortex. **B** The interaction networks between cell pairs identified in PN, PG, and PQ, respectively. **C** Identification of interactions of important ligand-receptor pairs in PN, PG, and PQ, respectively. The tail of the arrow represents the ligand, the direction represents the receptor. The thicker the line, the stronger the interaction.


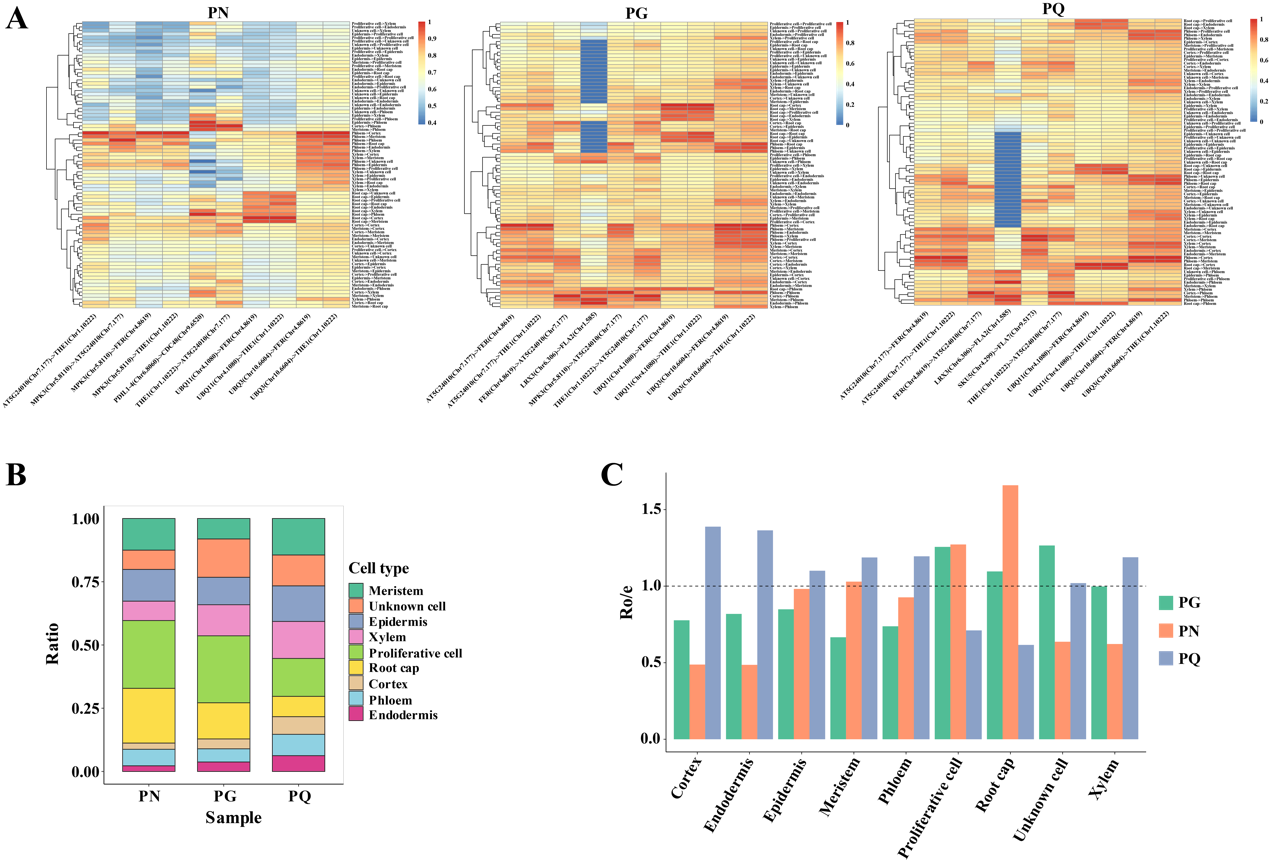


**Figure S13. A** The top 10 ligand-receptor pairs of PN, PG, and PQ indicated different regulatory patterns existed in the three species. **B** The proportion of 9 cell types in PN, PG, and PQ, respectively. **C** Preference of each cell type in PN, PG, and PQ.


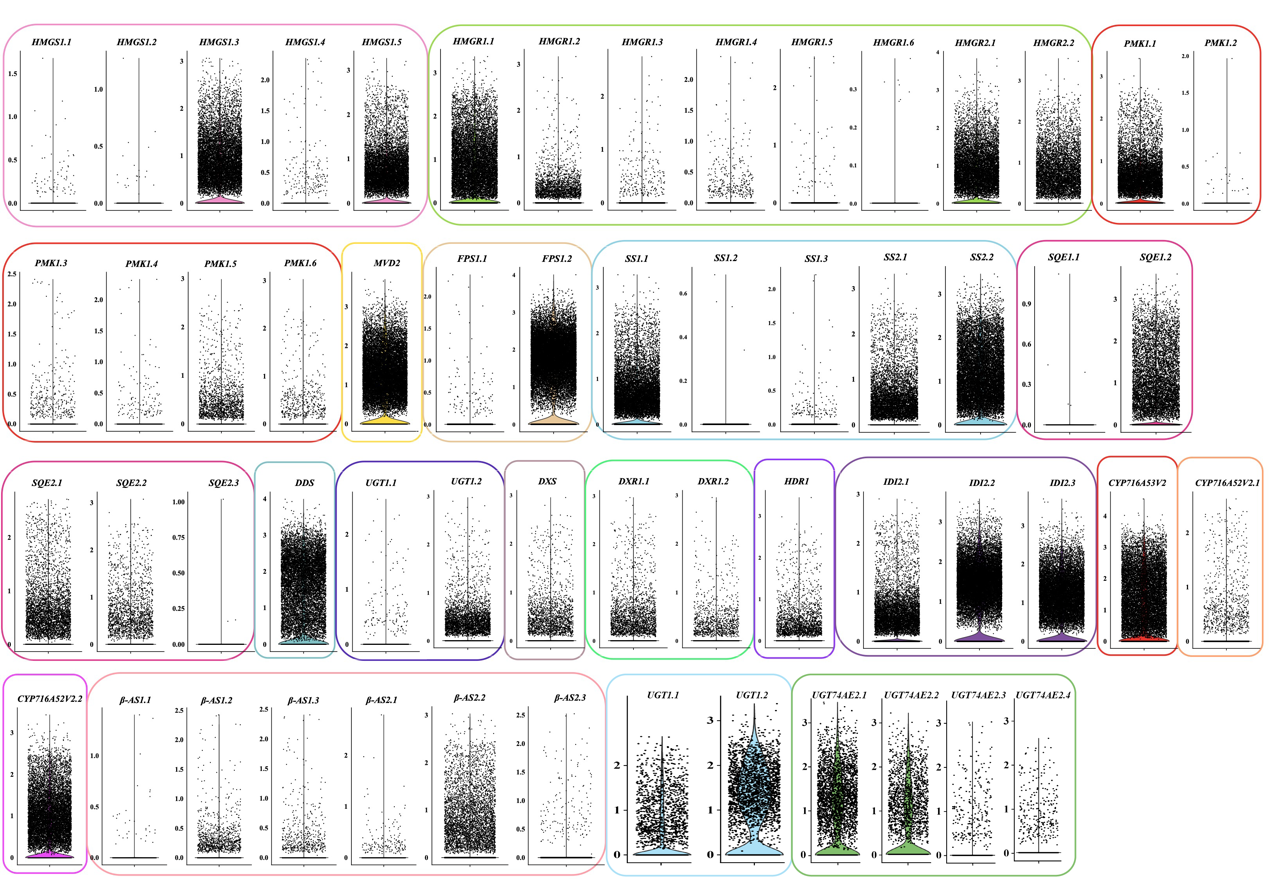


**Figure S14.** Cell expression percentage of key enzyme genes involved in ginsenosides biosynthesis pathways. VInPlot showing the main genes responsible for encoding key enzymes involved in ginsenosides biosynthesis. Each dot represents a cell, and the vertical axis indicates the expression proportion of corresponding gene in the cell.


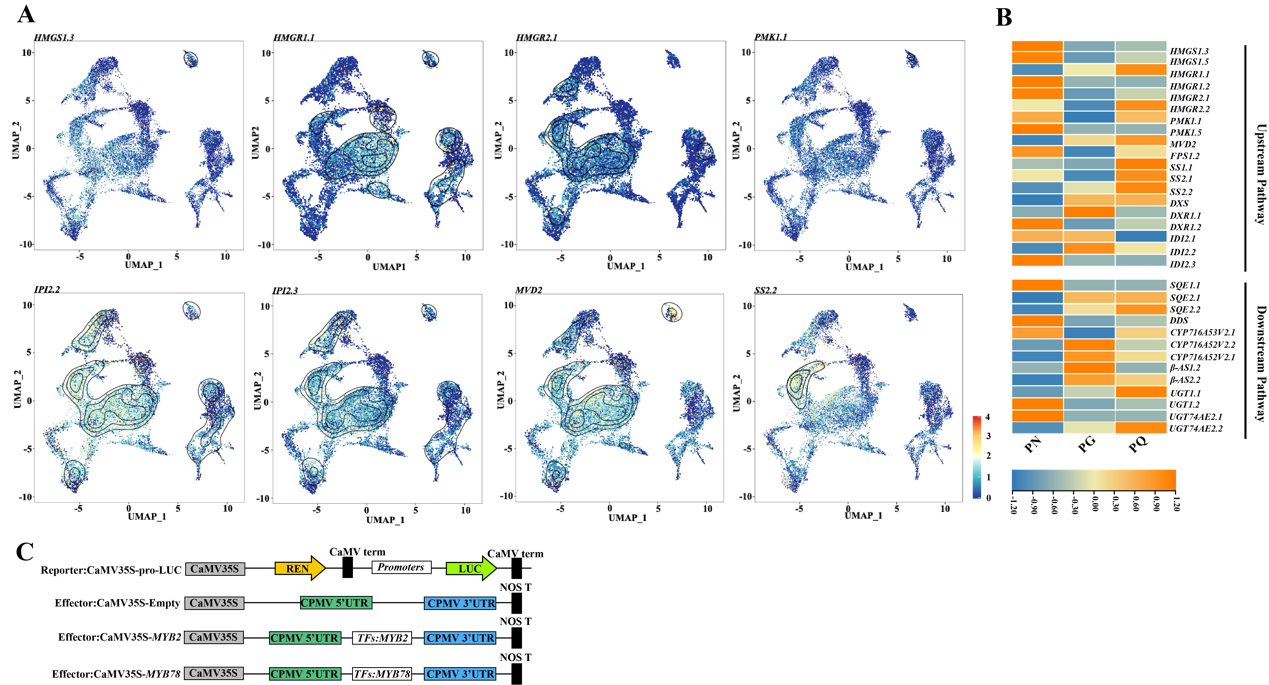


**Figure S15.** Expression patterns of key enzyme genes involved in ginsenosides biosynthesis pathways. **A** Expression profiles of genes involved in ginsenosides biosynthesis pathways in the three *Panax* species. **B** Expression profiles of key enzyme genes involved in ginsenosides biosynthesis pathways in nine cell types. **C** Overview of constructs prepared for dual-luciferase reporter assays. Promoter sequences of enzyme genes were ligated to the pGreenII 0800-LUC vector to produce the reporters. The effector was produced by inserting the TFs into the pGreenII-62-SK vector.


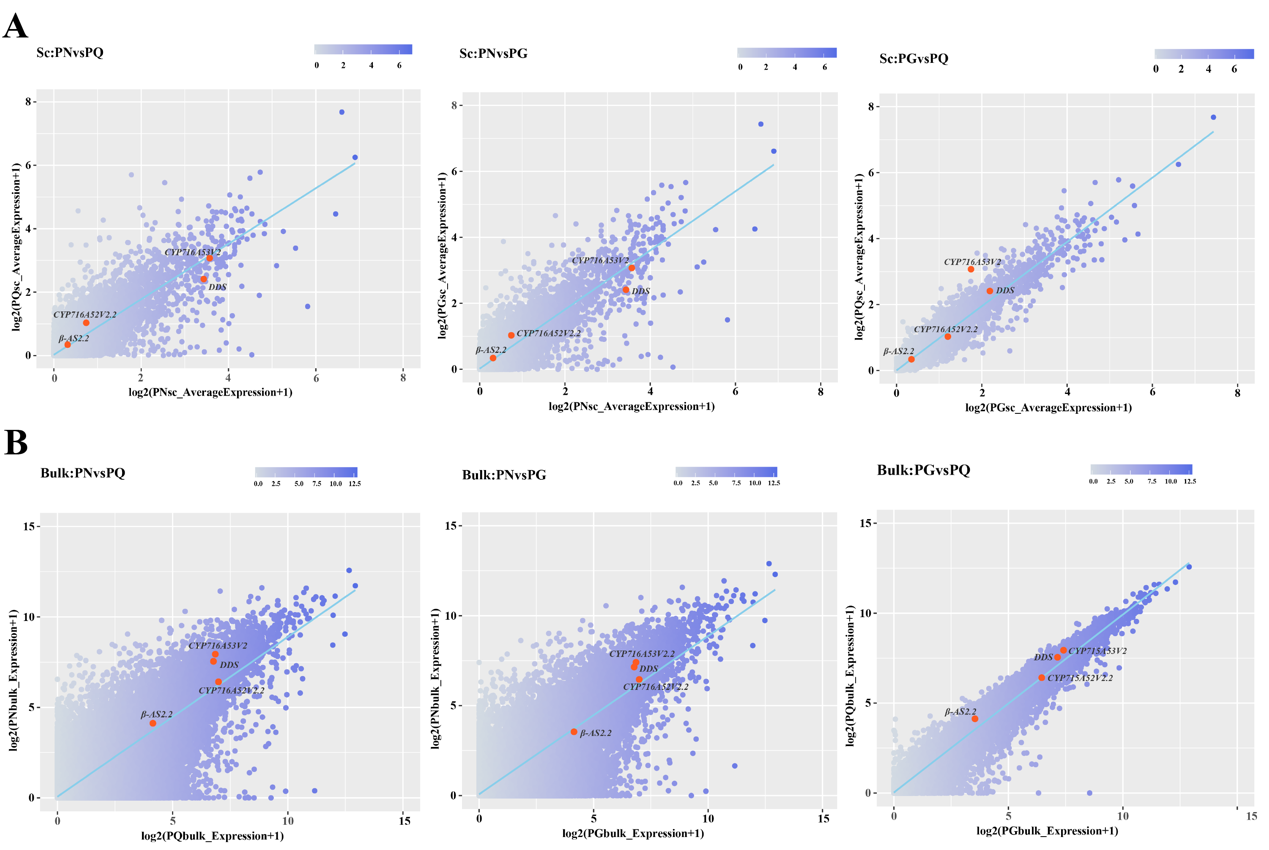


**Figure S16.** Correlation of gene expression between two different samples. **A** Correlation of gene expression based on scRNA-seq datasets between PN and PQ, PN and PG, as well as PG and PQ. **B** Correlation of gene expression based on bulk RNA-seq datasets between PN and PQ, PN and PG, as well as PG and PQ. Each dot represents a gene. For each gene, log2-transformed (RPM +1) values are plotted against each other. Color represents the expression level of the gene, with darker colors indicating higher expression levels. Red dots highlight four key downstream genes.


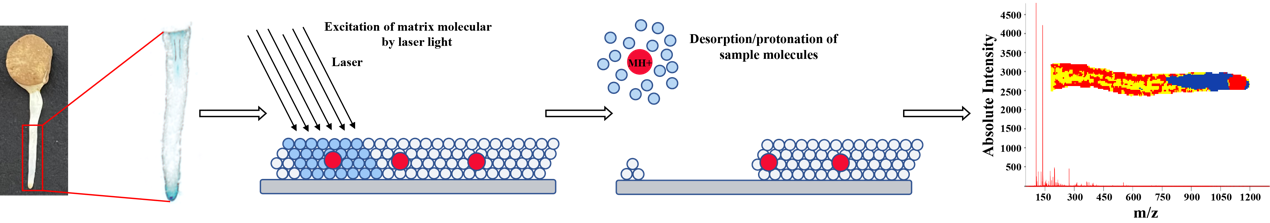


**Figure S17.** Sample preparation and MSI data generation. Sampling and sectioning method for MALDI2-MSI detection and MSI analysis generated a differentially colored map comprised of 3,214 points.


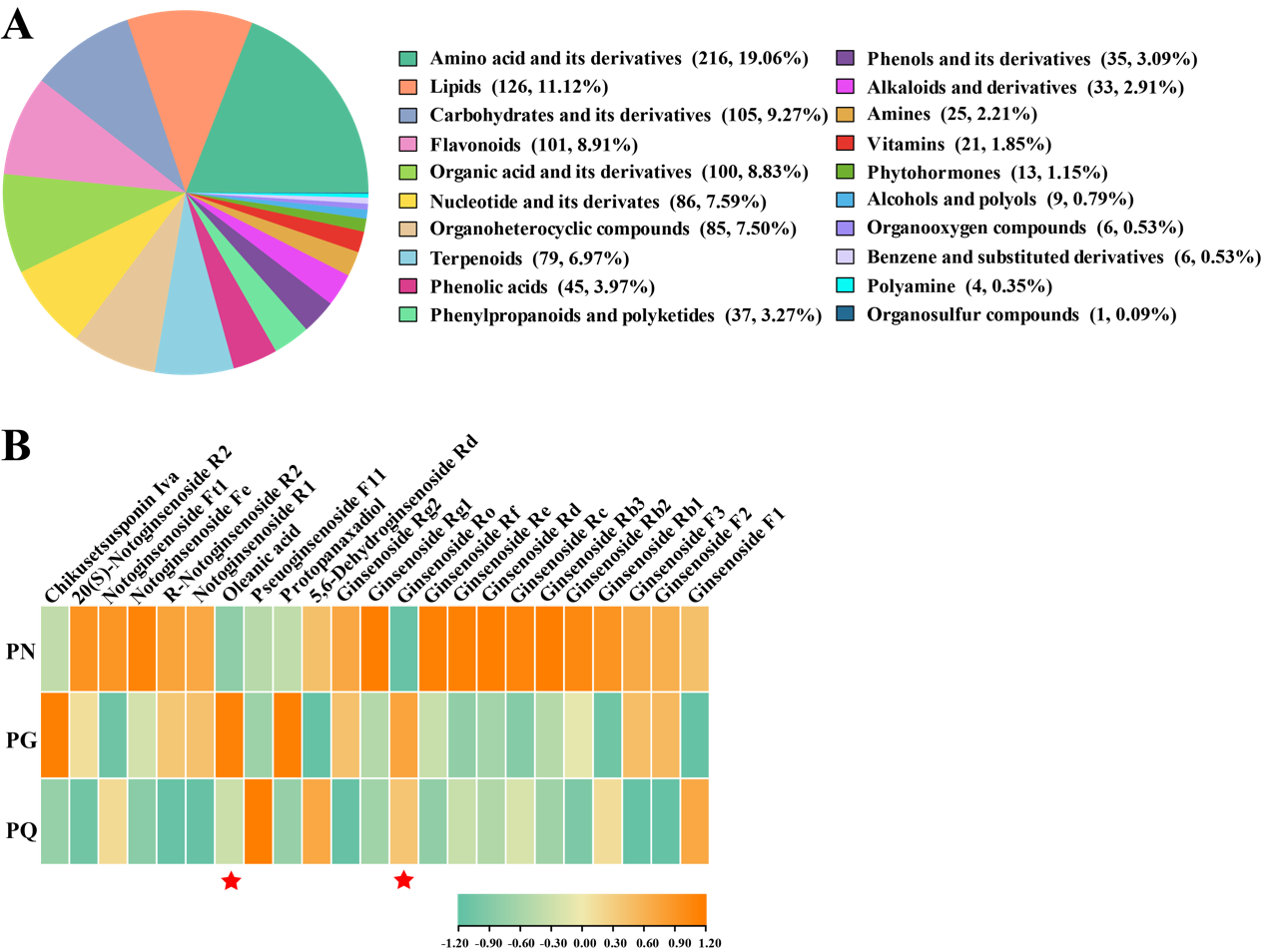


**Figure S18.** Metabolites identified from three *Panax* species through LC-MS/MS detection. **A** Chemical classification and proportion of all identified metabolites using LC-MS/MS. **B** Heatmap showing the content of 23 ginsenosides in PN, PG, and PQ.


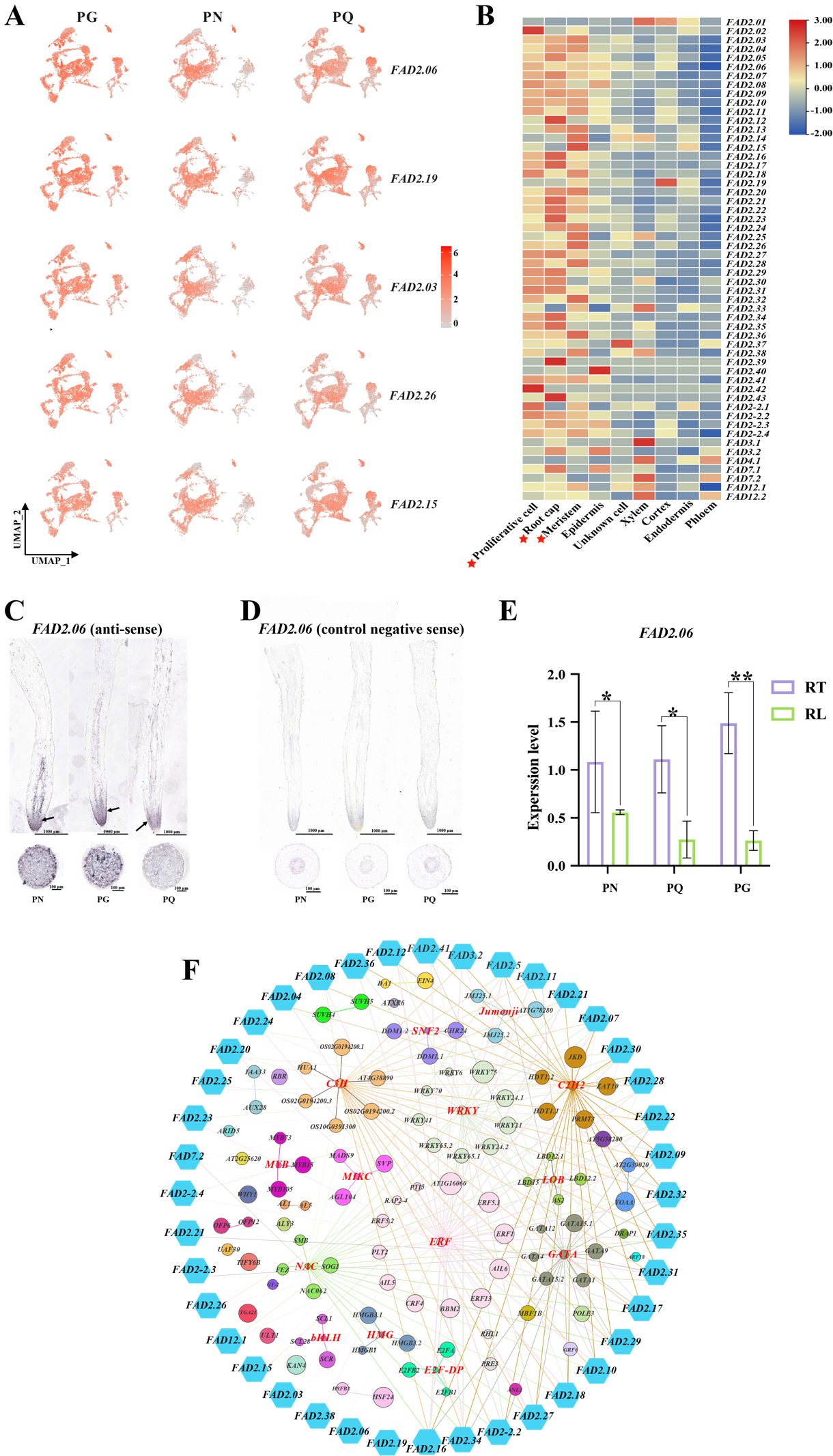


**Figure S19.** Expression patterns of the *FAD* gene family and related transcription regulation networks mediated by transcription factors. **A** Expression patterns of the top five *FAD* genes with average expression levels in PG, PN, and PQ, respectively. **B** Heatmap displaying the expression profiles of the *FAD* gene family across 9 cell types. Each row represents an FAD gene, and the expression levels within each row have been standardized to highlight the pattern of expression changes. Higher expression levels are indicated by redder colors. **C** Longitudinal and cross-sectional images from RNA *in situ* hybridization of the *FAD2.06* gene. **D** Longitudinal and cross-sectional images from control experiments of RNA *in situ* hybridization for the *FAD2.06* gene. **E** RT-qPCR validation results for the expression pattern of *FAD2.06* gene. **F** Correlation analysis between the *FAD* gene family and transcription factors. Transcription factors are represented by circles, and *FAD* genes by hexagons. Lines indicate correlations between TF family and *FAD* genes (correlation cutoff ≥ 0.6), with line width reflecting correlation strength. Circle size indicates the number of *FAD* genes regulated by corresponding TF, with larger diameters representing a higher number of regulated genes. Only correlation involving families with more than 5 TFs are shown.
